# Aspartoacylase promotes the process of tumour development and is associated with immune infiltrates in gastric cancer

**DOI:** 10.1186/s12885-023-11088-7

**Published:** 2023-06-30

**Authors:** Yalin Han, Xuning Wang, Maolin Xu, Zhipeng Teng, Rui Qin, Guodong Tan, Peng Li, Peng Sun, Hongyi Liu, Li Chen, Baoqing Jia

**Affiliations:** 1 Department of General Surgery, The First Medical Centre, Chinese PLA General Hospital, No. 28, Fuxing Road, Haidian District, Beijing, 100853 China; 2 The Air Force Hospital of Northern Theater PLA, Shenyang, 110042 China; 3 Department of Gastroenterology, The 305 Hospital of PLA, Beijing, 100017 China; 4 Air force medical center of PLA, Beijing, 100142 China; 5 Department of Oncology, Fifth Medical Center of Chinese PLA General Hospital, Beijing, 100071 China; 6 Department of Oncology, PLA Rocket Force Characteristic Medical Centre, Beijing, 100088 China

**Keywords:** Aspartoacylase, Gastric cancer, Prognostic biomarker, Immune infiltrates, Survival

## Abstract

**Background:**

Aspartoacylase (ASPA) is a gene that plays an important role in the metabolic reprogramming of cancer. However, the clinical relevance of ASPA in gastric cancer (GC) has not been demonstrated.

**Methods:**

The link between ASPA and the clinical features of GC was determined using two public genomic databases. The multivariate Cox proportional hazard model and generalised linear regression model were applied to examine whether the ASPA level is associated with the prognosis and other pathological factors. In addition, the role of specific genes in the infiltration of immune cells in the setting of GC was investigated using a further immunological database. The expression level of various proteins was detected using a western blotting assay. Transwell and methyl thiazolyl tetrazolium tests were applied for the detection of cellular invasion and proliferation, with small hairpin ribonucleic acid used to knockdown ASPA.

**Results:**

According to the multivariate Cox regression results, the down-regulated ASPA expression is a distinct prognostic factor. Furthermore, ASPA has significant positive correlations with the infiltration of immune cells in GC lesions. Compared to the non-cancer tissues, the GC tissues had a significantly lower level of ASPA expression (*p* < 0.05). Using knockdown and overexpression techniques, it was demonstrated that ASPA affects the capacity of cell lines for GC to both proliferate and invade.

**Conclusion:**

Overall, ASPA could promote the occurrence and development of GC and presents a promising predictive biomarker for the disease since it is favourably connected with immune infiltrates and negatively correlated with prognosis.

**Supplementary Information:**

The online version contains supplementary material available at 10.1186/s12885-023-11088-7.

## What is already known on this topic

The enzyme known as aspartoacylase (ASPA), which is found in the cytosol, catalyses the breakdown of n-acetylaspartate into aspartate and acetic acid, with the ASPA mutations leading to substantial increases in N-acetylaspartic acid concentrations in the brain, resulting in Canavan disease. The association between ASPA and cancer remains poorly understood.

## What this study adds

This research discovered that tumour tissues have lower levels of ASPA expression. The knockdown and overexpression of ASPA was found to affect the proliferation and invasion ability of gastric cancer (GC). Overall, ASPA was confirmed to be adversely correlated with the prognosis of GC and positively correlated with the immune infiltration of the disease, and could present a useful indicator in clinical practice.

## How this study might affect research, practice or policy

This research confirmed for the first time that ASPA may influence the development of GC and could be used as a predictor of the disease’s prognosis. It provides a new treatment idea for the potential future treatment of GC.

## Introduction

Gastric cancer (GC) is currently the third leading cause of cancer-related death and one of the top five most common cancers in the world [1]. Genetic and environmental variables both impact GC, which is a complex and polygenic disease [2]. Important risk factors include inherited genetic factors, high-risk food intake, alcohol abuse, smoking and *Helicobacter pylori* infections [3]. Although there exist many treatments for GC, including surgery, chemotherapy, targeted therapy and immunotherapy, the prognosis remains poor [4, 5]. Gastric cancer is a highly aggressive type of cancer and its heterogeneous character significantly affects both its incidence and its course of development [6]. However, the basic mechanism behind the onset and progression of GC remains poorly understood. Therefore, exploring the genetic characteristics of GC in depth is a crucial strategy for better understanding the pathogenesis of the disease.

One of the main features of cancer is metabolic reprogramming, which also drives the change in tumour cells, potentially exhibiting a variety of biological traits. Aspartoacylase (ASPA), a cytosolic lipogenic enzyme, catalyses the transformation of N-acetylaspartic acid (NAA) into aspartate and acetate [7], which occurs particularly in the brain [8]. Canavan disease is induced by mutations in ASPA, which significantly increase the concentration of NAA in the brain [9, 10]. The correlation between ASPA and cancer remains largely unknown, with a lack of evidence demonstrating the key role of ASPA in the progression of prostate cancer and breast cancer [8, 11]. Furthermore, it is unclear whether ASPA affects the prognosis and clinicopathological status of GC. Therefore, in this study, clinical samples, cell lines and bioinformatic techniques are used for the validation of the prognostic performance of ASPA in GC and to identify molecular markers for future cancer treatments.

## Materials and methods

### Clinical samples

Five individuals who had their GC tumours surgically removed between January 2017 and December 2021 at the First Medical Centre of the Chinese PLA General Hospital provided the surgical specimens. Our local institutional review board (the Ethics Committee of the Chinese PLA General Hospital) gave its approval for the utilisation of the clinical samples (reference number: S2020-326-01). All patients were made aware of the trial and gave their informed consent.

### Aspartoacylase data acquisition

The ASPA expression differences between tumour and control tissues were investigated using gene expression profiling interactive analysis (GEPIA) [12]. In addition, GEPIA was used for examining the role of ASPA in survival analysis. Various XENA (http://xena.ucsc.edu) datasets from The Cancer Genome Atlas (TCGA), including genomic data and clinical information, were obtained [13]. The Kaplan–Meier plot was utilised to verify the prognostic value of ASPA using the Gene Expression Omnibus (GEO) dataset [14].

### Survival analysis of aspartoacylase

The association between ASPA expression and various prognosis and clinicopathologic variables was examined using multivariate Cox proportional regression. The patients with GC were equally split into two groups and their Kaplan–Meier curves were examined. The relationship between ASPA and the clinical variables was then examined using a general linear regression model.

### Relationship between immunological infiltrates and aspartoacylase expression

The connection between ASPA and immunological infiltrates was examined using TIMER, an online tool for analysing immune infiltrates in TCGA samples (https://cistrome.shinyapps.io/timer/) [15]. Specific gene modules were used to examine the infiltration in tumour tissues, which included innate immune cells, antigen-processing cells and specific immune cells [16].

### Analysis of gene set enrichment

To assess the significance of a candidate gene and the alterations between two biological states, a computer-based approach known as gene set enrichment analysis was used [17].

The nominal *p*-value and the normalised enrichment score were used to identify the enriched pathways in each phenotype [18]. If the false discovery rate of a gene set was < 0.05, the set was considered statistically significant.

### Cell culture

The cell lines, HEK293T, AGS and BGC-823, were used in this investigation, with all the cell lines bought from the NCACC in China and kept at 37 °C in humidified incubators under a 5% carbon dioxide atmosphere. The Dulbecco’s modified Eagle medium–high-glucose medium used for all cell lines was supplemented with 10% fetal bovine serum (FBS) and 100 mg/mL of penicillin-streptomycin-glutamine (Gibco) [19].

### Lentivirus production and infection

The plasmid containing small hairpin ASPA (shASPA) was constructed in pLKO vector and the small hairpin ribonucleic acid (shRNA) sequences were as follows: shASPA-1: AATCAGATAAACGTAGCAGGG and shASPA-2: ATGGGTTCCTCCAAAGATAGC. To produce lentiviruses, the appropriate customised Lenti-EF1-puro plasmids were co-transfected in the HEK293T cells in the ratio of 5:1:5 in mass with the psPAX2 vector (for packing, Addgene) and pCMV-VSV-G (for enveloping, Addgene) (ng). Fresh media was added following transfection for 24 h to create a virus-containing conditioned medium. After 72 h, the virus-containing medium was collected and, if required, concentrated. For lentiviral infection, 0.5–1 mL of the virus-containing medium or 0.1–0.5 mL of virus concentrate were added to the cell culture for 48 h. The culture was reset after 48 h and a specific selection of medication was introduced to determine the resistance [19].

### Western blotting analysis

Passive lysis buffer (25 mM Tris-HCl, 150 mM NaCl, 0.5% CA630) containing a protease inhibitor cocktail was used to lyse the cells (Roche). The cells were boiled for five min in sodium dodecyl sulphate loading buffer before being lysed. Here, ASPA rabbit mAb (1: 1000; ab154503, Abcam) and GAPDH rabbit mAb (1: 1000; 10494-1-AP, Proteintech) were used as the main antibodies for the western blotting analysis, while all secondary antibodies (7074, Cell Signaling Technology) were employed at a 1:5000 dilution. A chemiluminescent substrate kit was purchased from Tanon, with Image J software used to quantify the findings [19].

### Cell transwell assay

In the top chamber, 2000 AGS and BGC-823 cells were plated per well, while the bottom compartment was filled with complete media containing 20% FBS. Following this, cells that had entered the chamber were fixed for 10 min using 4% paraformaldehyde (Solarbio), with washing using PBS performed three times to clean the wells. Colonies were stained for 5 min using Solarbio’s Crystal Violet Regent. The number of cell masses was counted under a Nikon microscope [19].

### 10 Methyl thiazolyl tetrazolium assay

A total of 1,000 cells were seeded per well in triplicates on 96-well plates, with the cells then incubated for 1–10 days. The plate was incubated at 37 °C for a further 4 h after methyl thiazolyl tetrazolium (MTT; thiazolyl blue tetrazolium, Sigma) was added to each well at a final concentration of 0.5 mg/ml. Dimethyl sulfoxide was added to each well and 100 μL of the media was withdrawn after the incubation. The test-ready plate was then tested at OD490 using a Biotek Synergy H1 microplate reader. Growth curves were created using OD490 values broken down by days [19].

### 11 colony formation assay

A number of AGS and BGC-823 cells were seeded in six-well plates at 2,000 cells/well on the third day after infection and were then cultured for 14 days to form colonies. The cells were subsequently treated by washing with PBS, fixing in 4% paraformaldehyde for 15 min, staining with 0.5% crystal violet for 1 h and washing three times using ddH^2^O before they were photographed using a digital camera.

### 12 statistical analysis

The statistical analysis was conducted using R-3.6.0, with the R package [20] used to draw a survival curve. A *p*-value of 0.05 or less was used as the cut-off threshold. Image J’s Analyze→Gels→Plot Lanes function was used to evaluate the western blot test findings and to perform image quantification. The experimental data were expressed in terms of mean and standard deviation, with a two-tailed Student’s *t*-test used for the statistical analysis. For the MTT assay, the two-way analysis of variance method was used for the statistical analysis, with the experimental findings expressed in terms of mean and standard deviation (*n* = 3 repetitions).

## Results

### Downregulation of aspartoacylase expression in gastric cancer

**Fig. 1:**
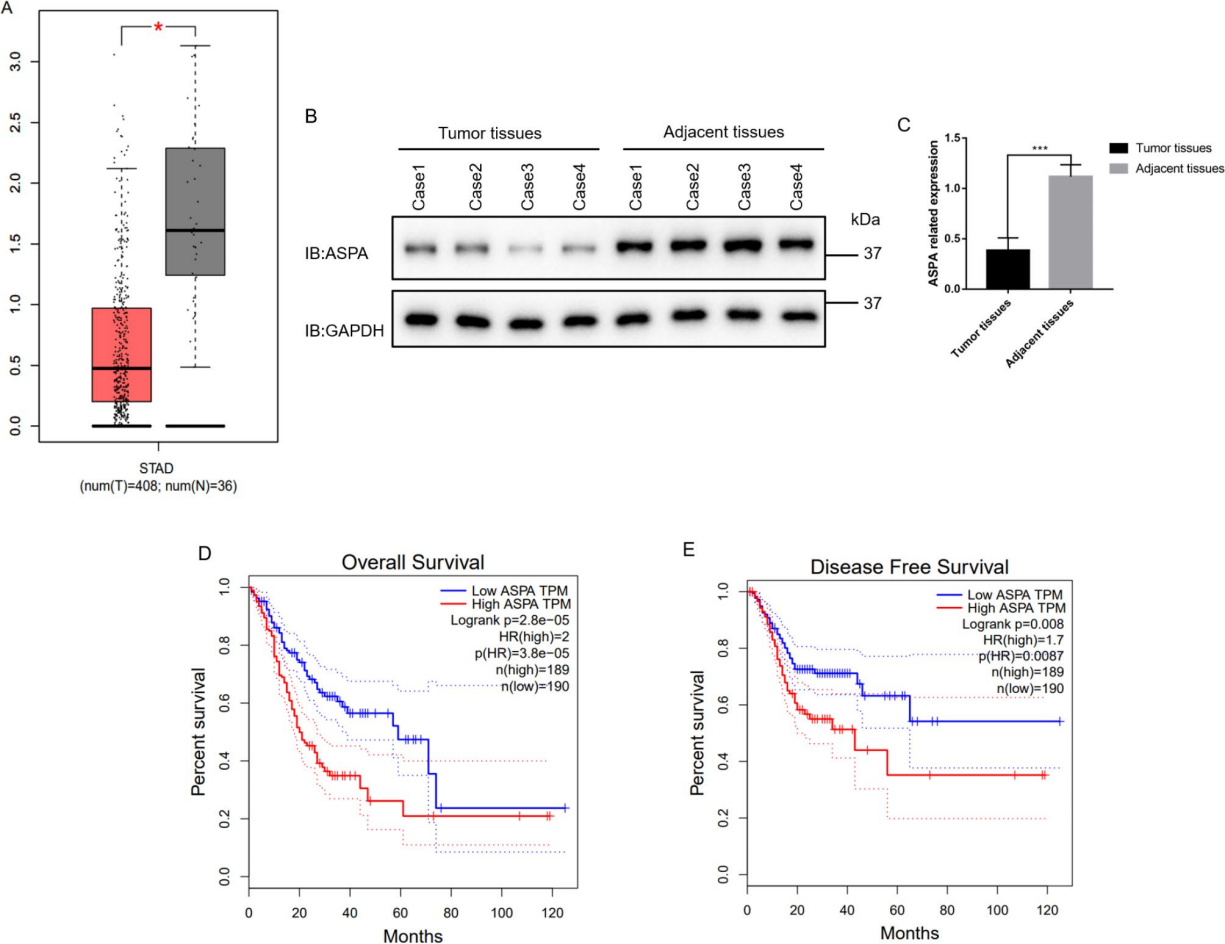
A. ASPA was downregulated in gastric cancer tissue. The num (T) and num (N) represent gastric cancer and similar nearby tissues, respectively. ASPA was decreased in tumor compared with paired samples. B, C. ASPA expression in clinical patient samples. D. The relationship between ASPA and overall survival. E. The relationship between ASPA and disease free survival. Error bar means ± SD, ***: *p* < 0.001

The ASPA was downregulated in the GC cohort. As Fig. 1A shows, compared to similar nearby tissues, the cancer tissues had a lower expression level of ASPA. The western blots presented a similar expression pattern of ASPA in the tumour and non-tumour tissues (*p* < 0001) (Fig. 1B C).

### Characterisation of the relationship between aspartoacylase and gastric cancer using bioinformatics analysis

We separated the samples into two equal groups according to the ASPA expression. The patients with low ASPA expression were more likely to survive than those with high ASPA expression (Fig. 1D).

The survival analysis also indicated that the disease-free survival time was significantly prolonged in the group with a lower level of ASPA compared to the patients who had a higher level of ASPA expression (Fig. 1E). The above results were obtained using the GEPIA database.

**Fig. 2:**
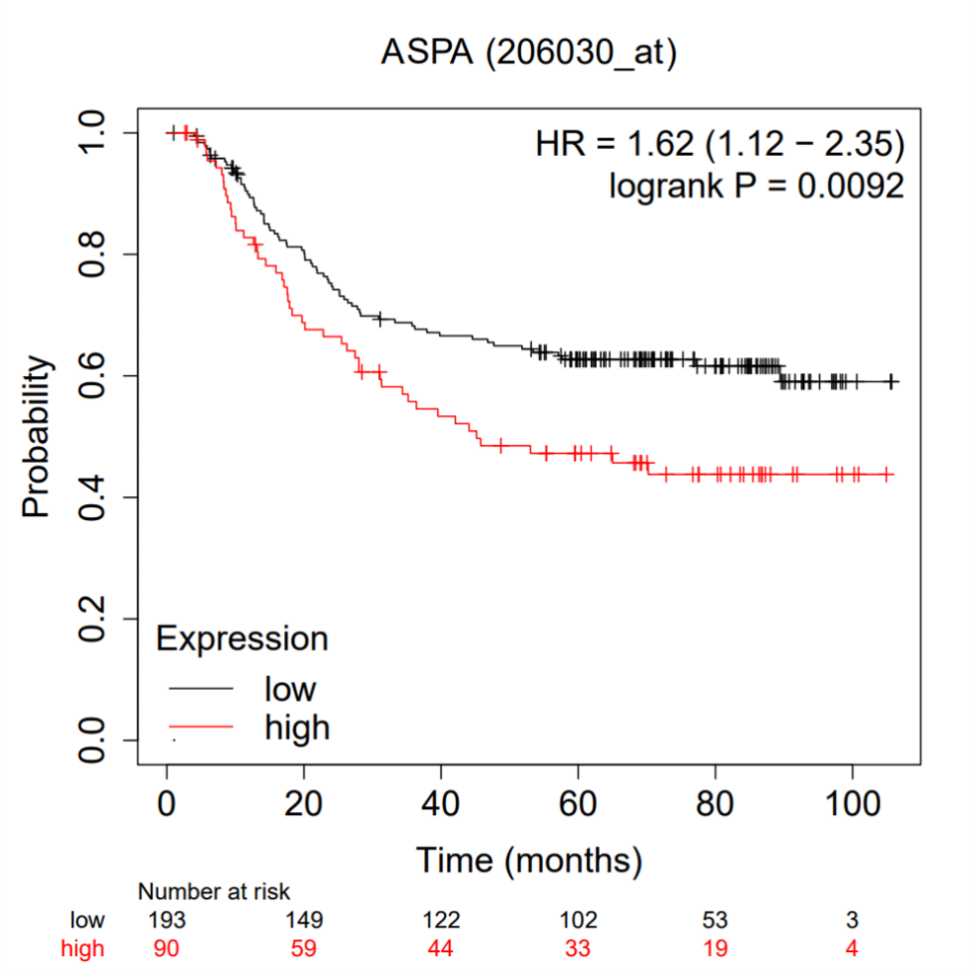
Patients with low expressed ASPA had significantly longer overall survival

Furthermore, GEO GC data were used for verification. The overall survival was considerably longer for the patients with low ASPA expression than for those with high expression (Fig. 2).

**Fig. 3:**
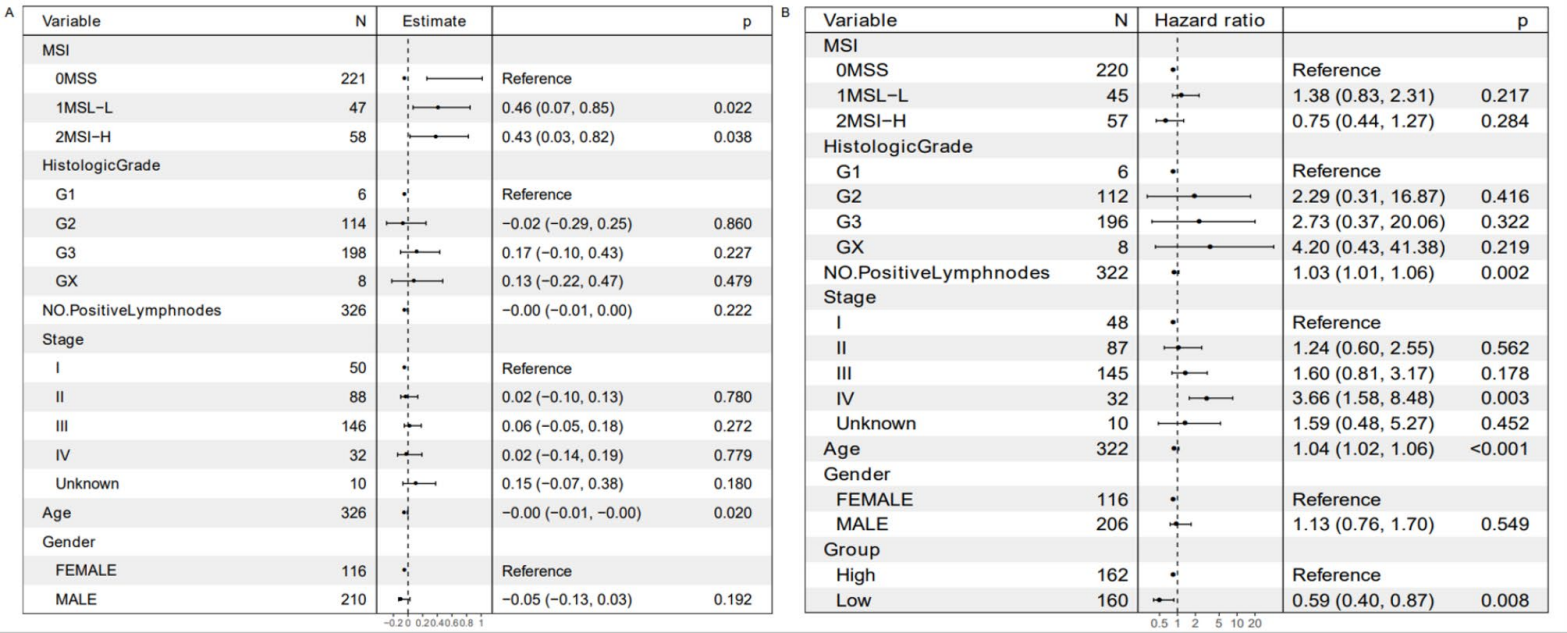
A. ASPA expression is significantly associated with microsatellite instability. B. Multivariate COX proportional hazard regression model showed ASPA was an independent prognosis biomarker

In addition, the relationship between ASPA and specific clinicopathologic factors was explored. As shown in Fig. 3, the general linear regression model indicted that there is a strong correlation between ASPA expression and microsatellite instability.

According to the multivariate Cox proportional hazard regression model, the survival of the GC patients was significantly correlated with ASPA expression. In this research, the number of positive lymph nodes served as an independent prognostic predictor (Fig. 3). Given the above findings, ASPA expression may present a useful predictive biomarker. The implication is that a favourable outcome for GC is substantially related to a lower expression of ASPA.

### Stimulation of the growth and invasion of gastric cancer by aspartoacylase

**Fig. 4:**
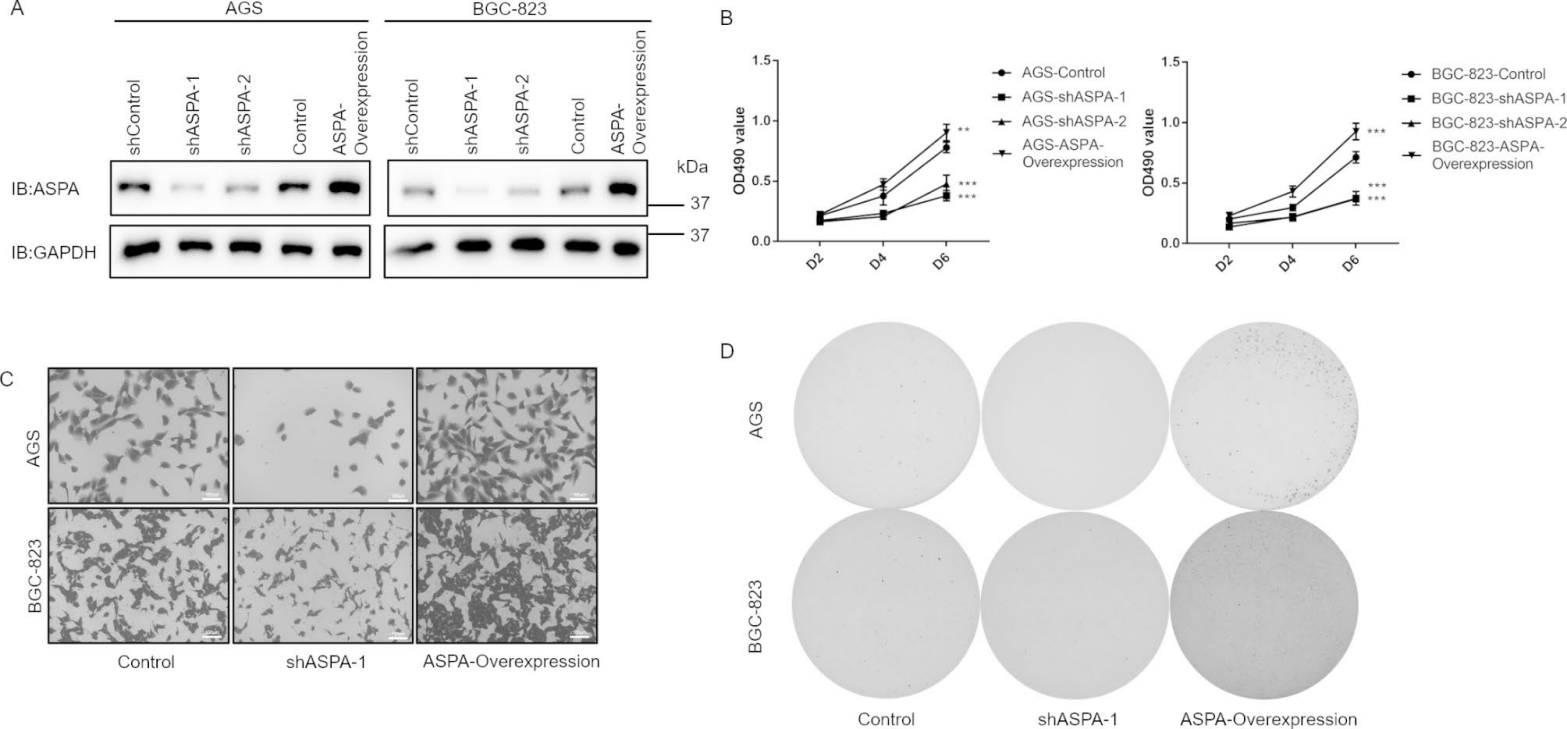
A. Knockdown and overexpression of ASPA using lenti-virus in AGS and BGC-823 cells. The knock-down and overexpression efficiency was determined by western blotting. B. MTT assay was used to detect the proliferation ability of AGS and BGC-823 cells. n = 3. Error bars mean ± SD, by two-way ANOVA analysis. C. Transwell assay was used to detect the invasion ability of AGS and BGC-823 cells. D. Colony formation assay was used to detect the proliferation ability of AGS and BGC-823 cells. ** :*p* < 0.01; *** :*p* < 0.001

Both knockdown and overexpressing ASPA stable cell lines were constructed using lentiviruses (Fig. 4A). The results indicated that a stable cell line was successfully constructed. The MTT experiment results revealed that ASPA knockdown dramatically decreased the proliferative potential of the GC cell lines (*p* < 0.001) (Fig. 4B). Following overexpression, the ASPA improved the ability of the GC cell lines to proliferate (*p* < 0.01) (Fig. 4B). The transwell test findings demonstrated that the suppression of ASPA greatly reduced the capacity of the GC cell lines to invade, with increased GC cell line invasion observed following ASPA overexpression (Fig. 4C). The knockdown of the ASPA reduced the clonogenic ability of both the AGS and BGC-823 cells, while conversely, the overexpression of ASPA increased the clonogenic ability of both types of cells (Fig. 4D). The above findings suggest that ASPA could have an impact on the capacity of GC cells for invasion and proliferation.

### Aspartoacylase expression in relation to immune infiltration in gastric cancer

**Fig. 5:**
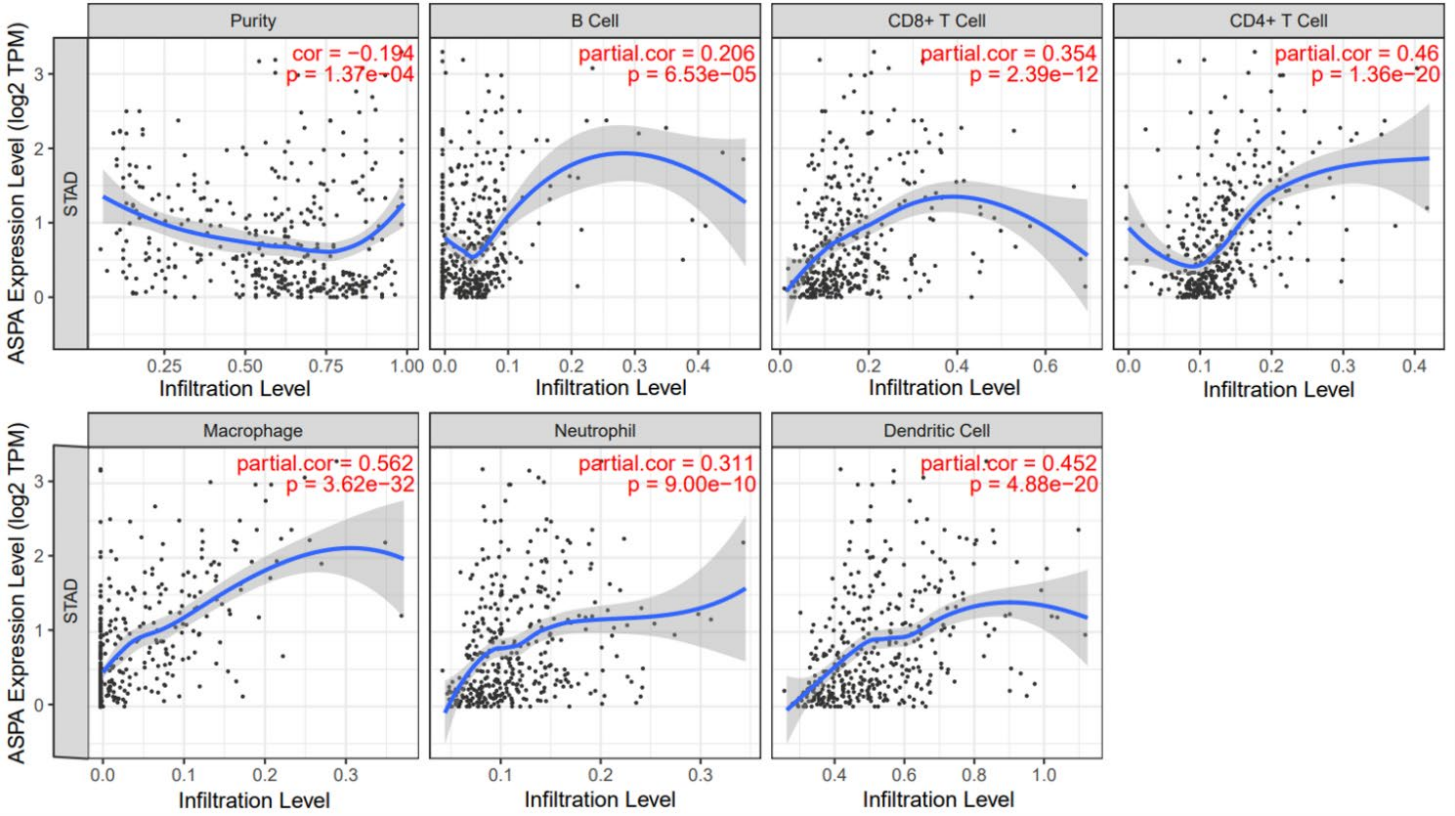
The expression of ASPA was significantly associated with B cell (r = 0.206, p = 6.53e-05), CD8 + T cell (r = 0.354, p = 2.39e-12), CD4 + T cell (r = 0.46, p = 1.36e-20), Macrophage (r = 0.562, p = 3.62e-32), Neutrophil (r = 0.311, p = 9.00e-10) and Dendritic cell (r = 0.452, p = 4.88e-20)

According to earlier study findings, tumour-infiltrating lymphocytes are regarded as an independent biomarker for predicting the prognosis of tumour patients who are the focus of immune therapy [21, 22]. As such, we aimed to confirm whether the ASPA expression in GC is connected to immune infiltration. There were significant correlations between the expression of ASPA and B cells (*r* = 0.206, *p* = 6.53e-05), CD8 + T cells (*r* = 0.354, *p* = 2.39e-12), CD4 + T cell (*r* = 0.46, *p* = 1.36e-20), macrophage (*r* = 0.562, *p* = 3.62e-32), neutrophil (*r* = 0.311, *p* = 9.00e-10) and dendritic cells (r = 0.452, *p* = 4.88e-20). In terms of GC, there was a negative correlation between ASPA levels and tumour purity (Fig. 5).

### Gene sets enriched in aspartoacylase expression phenotype

**Fig. 6:**
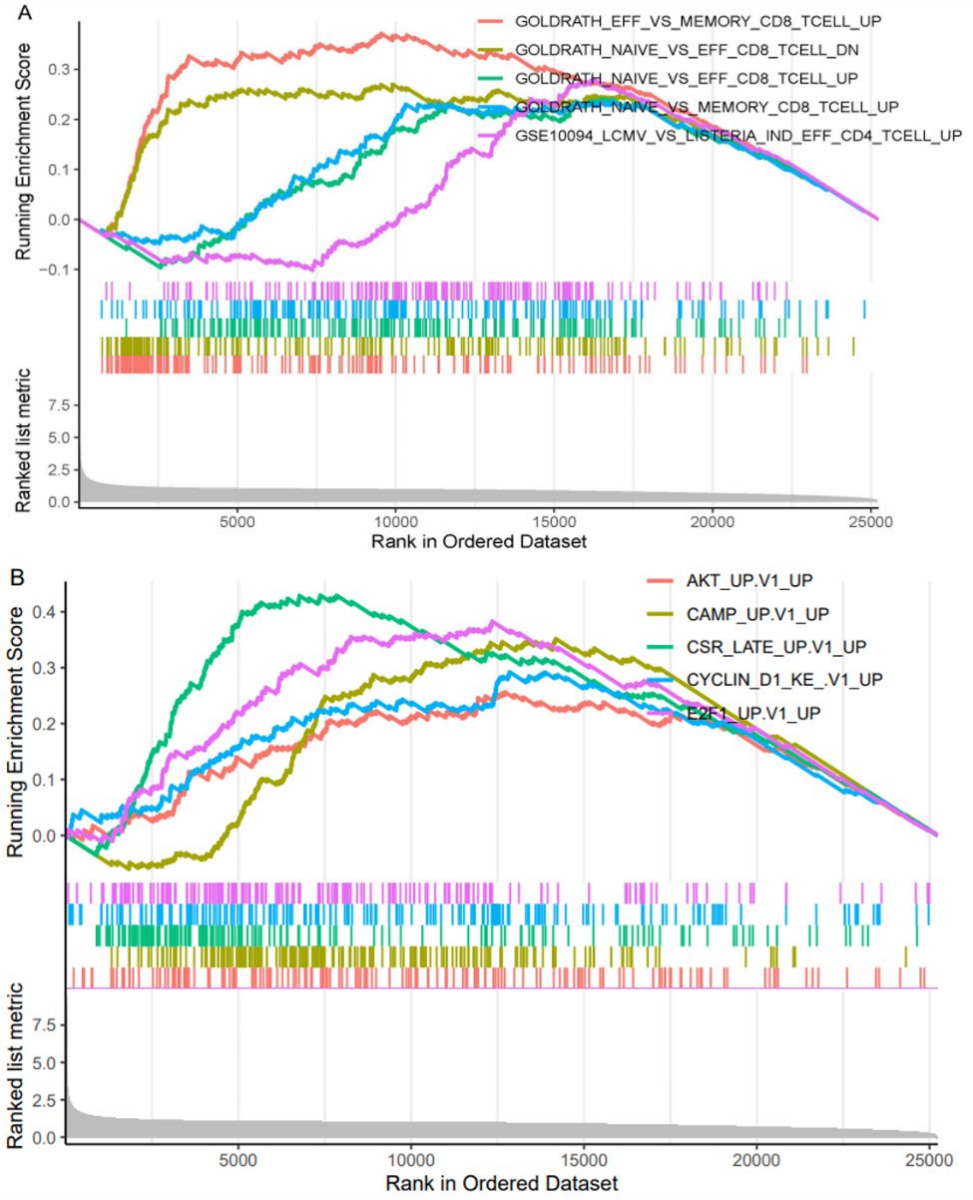
A. GSEA analysis revealed ASPA was involved in many important pathways. B. Top 5 pathways were graphed

Based on the expression of ASPA as discussed above, all the samples used were again separated into two groups. Only the top enriched paths are presented in Fig. 6 due to space restrictions. As the figure shows, ASPA was preferentially enriched in the pathways associated with protein kinase B (AKT), cyclic adenosine 3’, 5’-monophosphate (cAMP), class-switch recombination (CSR), cyclin D1 and E2F transcription factor 1 (E2F1).

## Discussion

The growth and proliferation of cancer cells depend on metabolic reprogramming [23]. Evidence indicates that metabolic reprogramming may become a new direction for tumour-targeted therapy [24]. A number of studies have highlighted the benefits of using therapeutic targets of the tumour-associated metabolic pathways for anticancer medicines, and recently, important functions of amino acid metabolism in cancer development and prognosis have been found [25, 26]. Meanwhile, ASPA is an essential metabolite during the metabolic reprogramming of cancer. Therefore, in this investigation, we sought to ascertain whether ASPA plays a role in the development of GC. Here, it was discovered that the ASPA expression was markedly reduced in the GC tissues compared to nearby tissues. Sun et al. [11] reported that ASPA expression was decreased in both the breast and prostate cancer samples compared to the control samples. Long et al. reported that ASPA expression is also lower in glioma than in normal tissues [27], while Tsen et al. [28] demonstrated that efficient targeting of ASPA may diminish glioma development. As a result, we hypothesised that ASPA may behave as a tumour-promoting factor as GC evolves. Both the proliferation and the invasion capacity of GC cancer cell lines found to be altered in the ASPA knockdown and overexpression experiment. In this study, it was also demonstrated that ASPA is a prognostic factor. Poor survival was predicted by higher ASPA levels.

Contradictory findings pertaining to ASPA have been found in terms of different cancer types, with, for example, the hazard ratio (HR) of GC found to be > 1 and the HR of liver cancer found to be < 1, indicating that the role of ASPA in the development of malignant tumours may be organ-specific (Figure S1). Figure S1 also shows two cancer types with a HR of significantly higher than 1 (risk factor): stomach adenocarcinoma (STAD) and lung squamous cell carcinoma (LUSC).

Gastric cancer is a major health problem worldwide, ranking fifth among the most common malignancies and the third leading cause of cancer-related deaths worldwide [29]. There are four pathological types of GC: adenocarcinoma, adenosquamous carcinoma, squamous carcinoma and carcinoid carcinoma, with 95% of GCs gastric adenocarcinomas. Therefore, early diagnosis is crucial to the prognosis of patients with gastric adenocarcinoma (STAD). Higher levels of aromatic amino acids in gastric juice have been reported in the early stages of cancer progression [30]. This implies that there is an association between GC and amino acid metabolism. However, studies have reported the prevalence of *H. pylori* babA, homB, aspA and sabA genes in Turkey and their association with clinical outcomes [31]. It is known that *H. pylori* is one of the important factors in the development of GC, which appears to weaken the association between ASPA and STAD. Further discussion on the relationship between ASPA and STAD is required.

Meanwhile, LUSC is the most common type of non-small cell lung cancer, accounting for around 30% of lung cancer cases. Microarray data analysis of LUSC tumours identified four gene expression subtypes (canonical, basal, primitive and secretory) [32]. It has been reported that the gene characteristics of abnormal expression of amino acid and fatty acid metabolism are critically related to the pathogenesis of LUSC [33].

In addition, our results indicated that ASPA expression is significantly associated with microsatellite instability. Emerging data has suggested that microsatellite instability status is an effective biomarker for predicting the efficacy of immunotherapy for GC [34], with previous studies revealing its prognostic role [35]. Previous research has also shown that a variety of processes, including gene mutation encoding important enzymes engaged in metabolic pathways, may affect the metabolism of cancer cells. However, MSI-high (MSI-H) gastric and/or colorectal tumours are the only types to have mutations in ASPA (MSI-low or MSI-stable cancers do not), indicating that ASPA is related to tumour heterogeneity [36] and has a close connection with microsatellite instability.

In addition, in the present study, it was confirmed that ASPA is significantly related to immune infiltration in GC using the TIMER dataset. According to previous research, immune infiltration plays a significant role in patient outcomes, while tumour-associated macrophage and neutrophil infiltration play a significant role in patient prognosis and tumour chemosensitivity [37]. The immune microenvironment in MSI-H tumours of colorectal cancer samples present significant infiltration of lymphocytes [38].

Overall, ASPA is a useful biomarker that merits further study in relation to GC due to the finding that the expression of ASPA is correlated with several different immune markers in GC. This finding suggests that ASPA may be involved in controlling immune cell infiltration in this type of cancer.

Our results further indicated that ASPA is preferably enriched in cAMP, CSR, E2F1, AKT and cyclin D1-related pathways. These pathways are essential to the development of GC. In fact, cAMP plays an important role in cellular responses to many hormones and neurotransmitters [39]. One study found an interaction between histamine and cAMP in the human GC cell line, hgt-1 [40]. In the present study, while no association between the CSR signalling pathway and GC was found, the opposite was the case for E2F1. In a recent study, E2F1 was found to induce terminal differentiation-induced ncRNA (TINCR) transcriptional activity and accelerate GC progression through activation of the related signalling axis [41]. Regarding AKT, it has long been recognised that it is required for cell growth, proliferation and survival. Aberrant activation of AKT is one of the most common molecular findings in human malignancies, including GC, and is believed to play an important role in cancer cell survival and chemoresistance. Combining phosphatidylinositol 3-kinase/AKT pathway inhibitors with chemotherapy has been successful in reducing the chemoresistance in GC cell lines [42]. It has been proposed that the overexpression of cyclin D2 (but not cyclin D1) is closely associated with GC [43], while some have reported that cyclin D1 overexpression is, in fact, associated with the disease. The relationship between cyclin D2 and GC requires further discussion [44].

Several studies have confirmed that the expression of ASPA is decreased in tumours (prostate cancer, glioblastoma, neuroblastoma) [8, 45, 46] and is correlated with poor prognosis (glioblastoma, neuroblastoma). Current evidence suggests that ASPA is related to cell cycle regulation [47] and may subserve a signalling function [48]. Through the aforementioned routes, ASPA may potentially have an impact on the incidence and growth of malignancies. As a result, we may have better knowledge of how amino acid metabolism contributes to the development of cancer and its clinical significance to GC.

## Conclusion

In conclusion, ASPA is associated with the occurrence and development of GC and affects the survival rate of patients with this disease. It was also observed that the expression of ASPA in GC is associated with immune infiltration. Our findings indicate a new possibility for the pathogenesis of GC, with ASPA potentially serving as an important regulatory factor and useful predictor of the immune infiltration of GC. Overall, the findings provide directions for further research and the treatment of GC. It is worth noting that altering amino acid metabolism remains an unexplored area in the current field of cancer research.

## Electronic supplementary material

Below is the link to the electronic supplementary material.

Supplementary Material 1

Supplementary Material 2

Supplementary Material 3

Supplementary Material 4

Supplementary Material 5

Supplementary Material 6

Supplementary Material 7

Supplementary Material 8

Supplementary Material 9

Supplementary Material 10

Supplementary Material 11

Supplementary Material 12

Supplementary Material 13

Supplementary Material 14

Supplementary Material 15

Supplementary Material 16

Supplementary Material 17

Supplementary Material 18

Supplementary Material 19

Supplementary Material 20

Supplementary Material 21

Supplementary Material 22

Supplementary Material 23

Supplementary Material 24

Supplementary Material 25

## Data Availability

All data generated or analyzed during this study are included in this published article. The data that support the findings of this study are available from the corresponding author, Baoqing Jia, upon reasonable request.
