# Supplementary material for: Aspartoacylase promotes the process of tumour development and is associated with immune infiltrates in gastric cancer
Source: BMC Cancer. 2023 Jun 30;23:604. doi: 10.1186/s12885-023-11088-7 (PMC10311717; doi:10.1186/s12885-023-11088-7)
Supplement: Supplementary file 1 — Supplementary Material 1 [file 12885_2023_11088_MOESM1_ESM.docx]

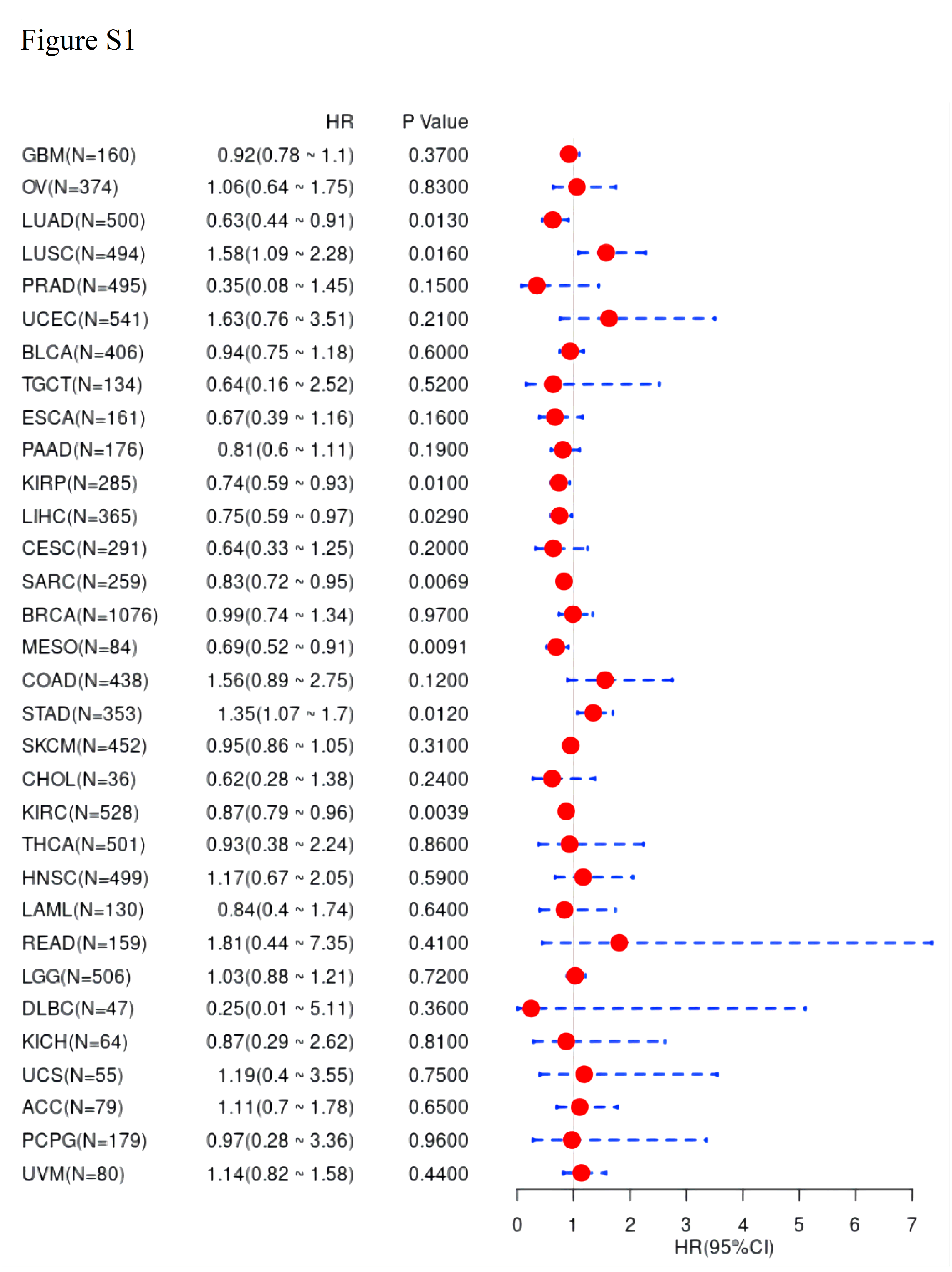


Figure S1. COX regression model of ASPA in an-cancer analysis. Hazard ratio (HR) variates suggested ASPA may be a risk factor or protective factor in different type cancers.
