# Supplementary figures and images for: Aspartoacylase promotes the process of tumour development and is associated with immune infiltrates in gastric cancer
Source: BMC Cancer. 2023 Jun 30;23:604. doi: 10.1186/s12885-023-11088-7 (PMC10311717; doi:10.1186/s12885-023-11088-7)

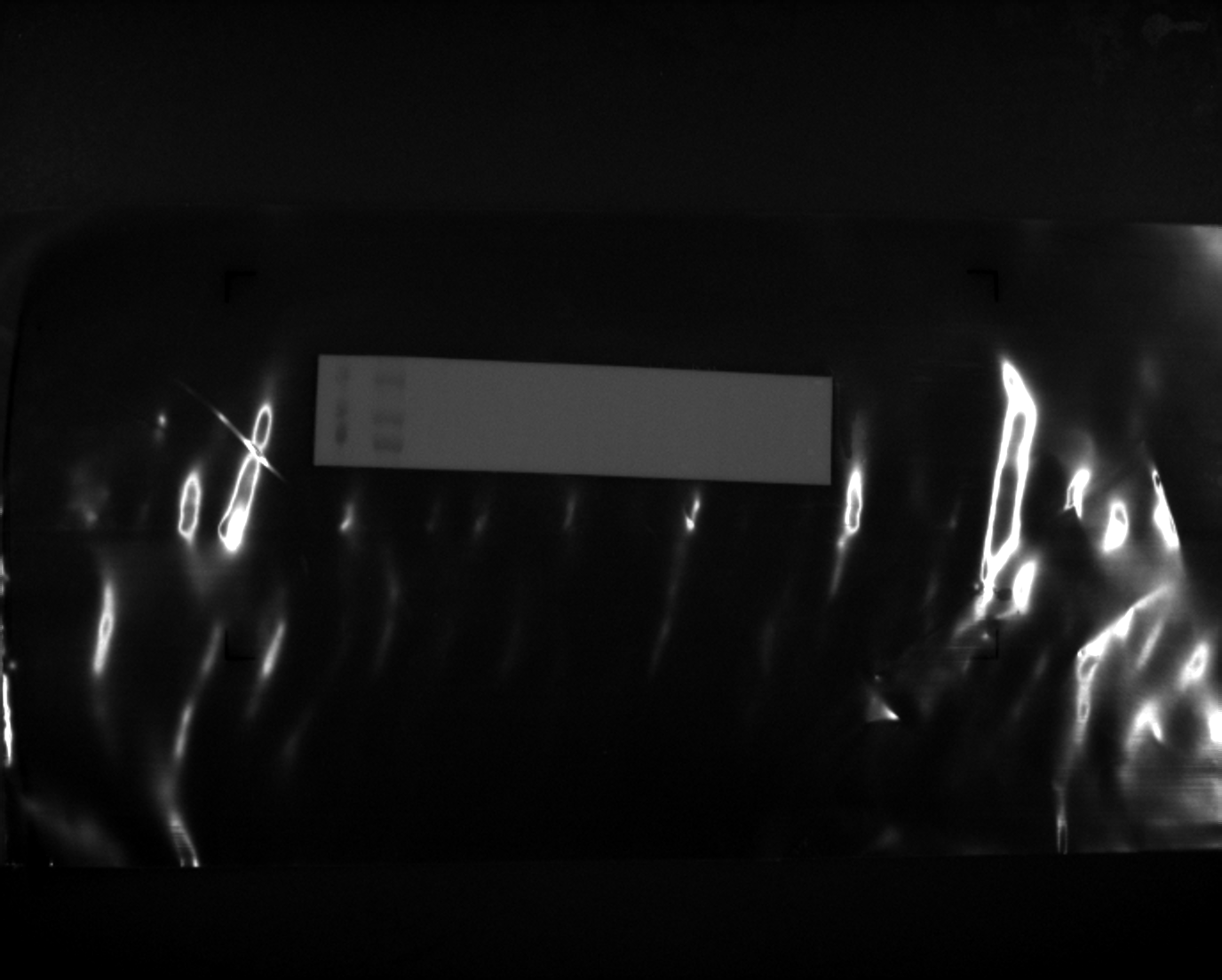

Supplement: Supplementary file 2 — Supplementary Material 2 [file 12885_2023_11088_MOESM2_ESM.tif]

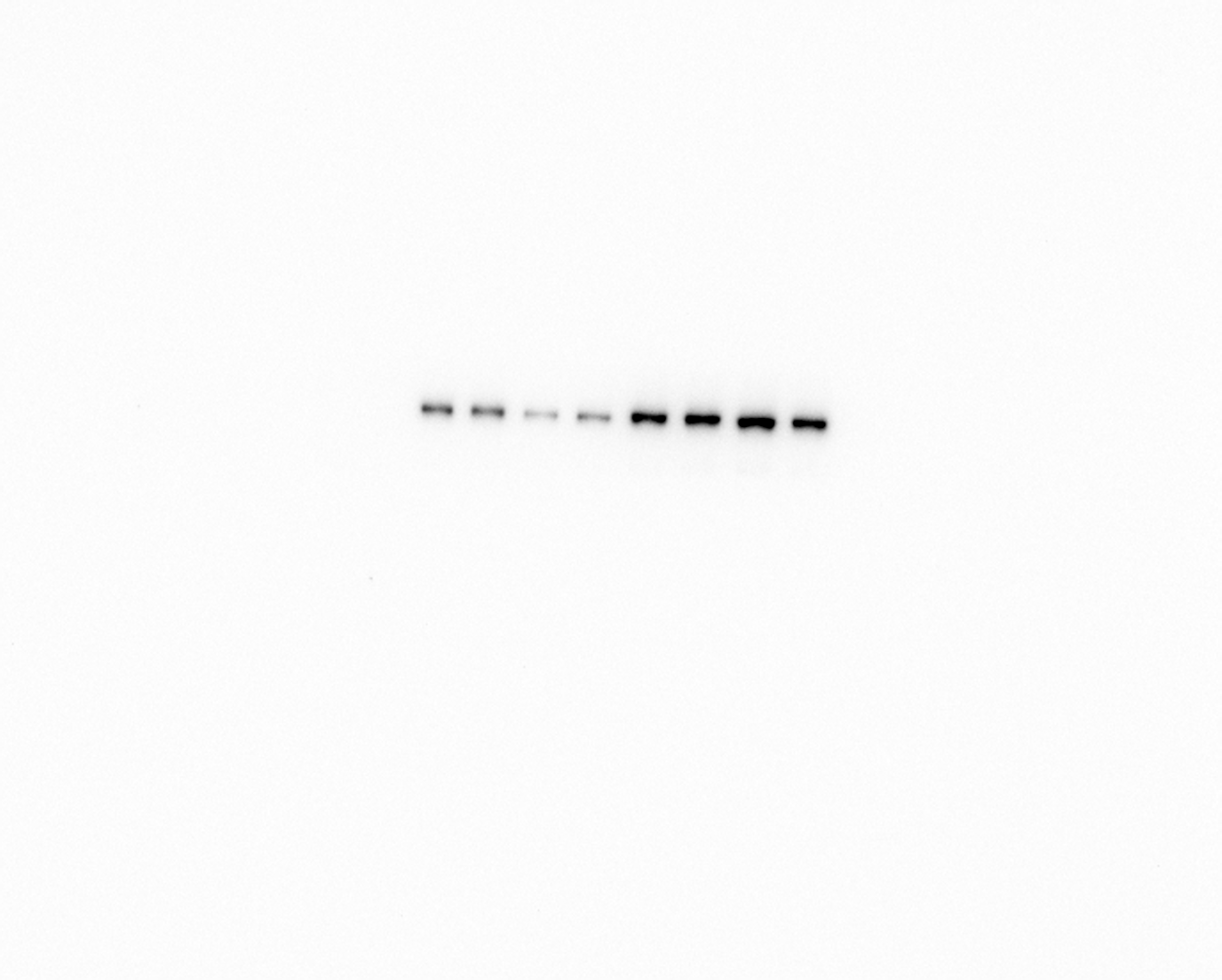

Supplement: Supplementary file 3 — Supplementary Material 3 [file 12885_2023_11088_MOESM3_ESM.tif]

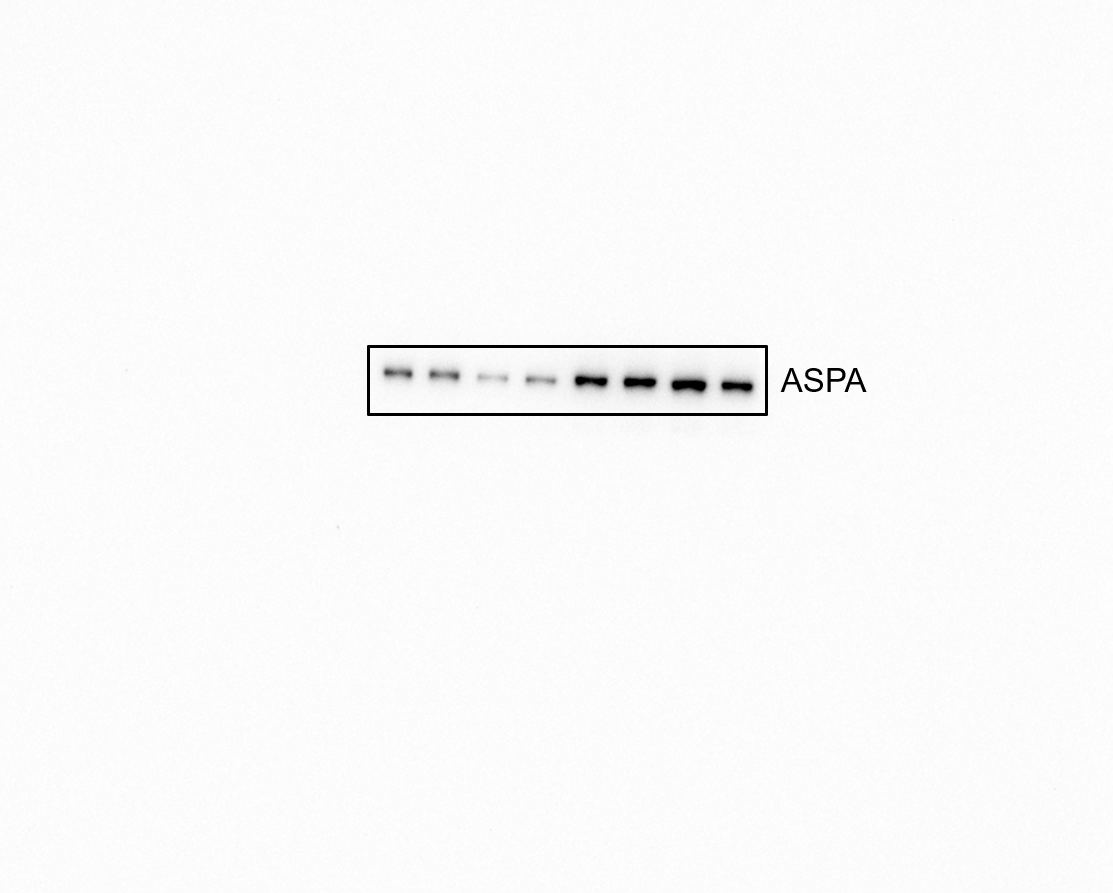

Supplement: Supplementary file 4 — Supplementary Material 4 [file 12885_2023_11088_MOESM4_ESM.tif]

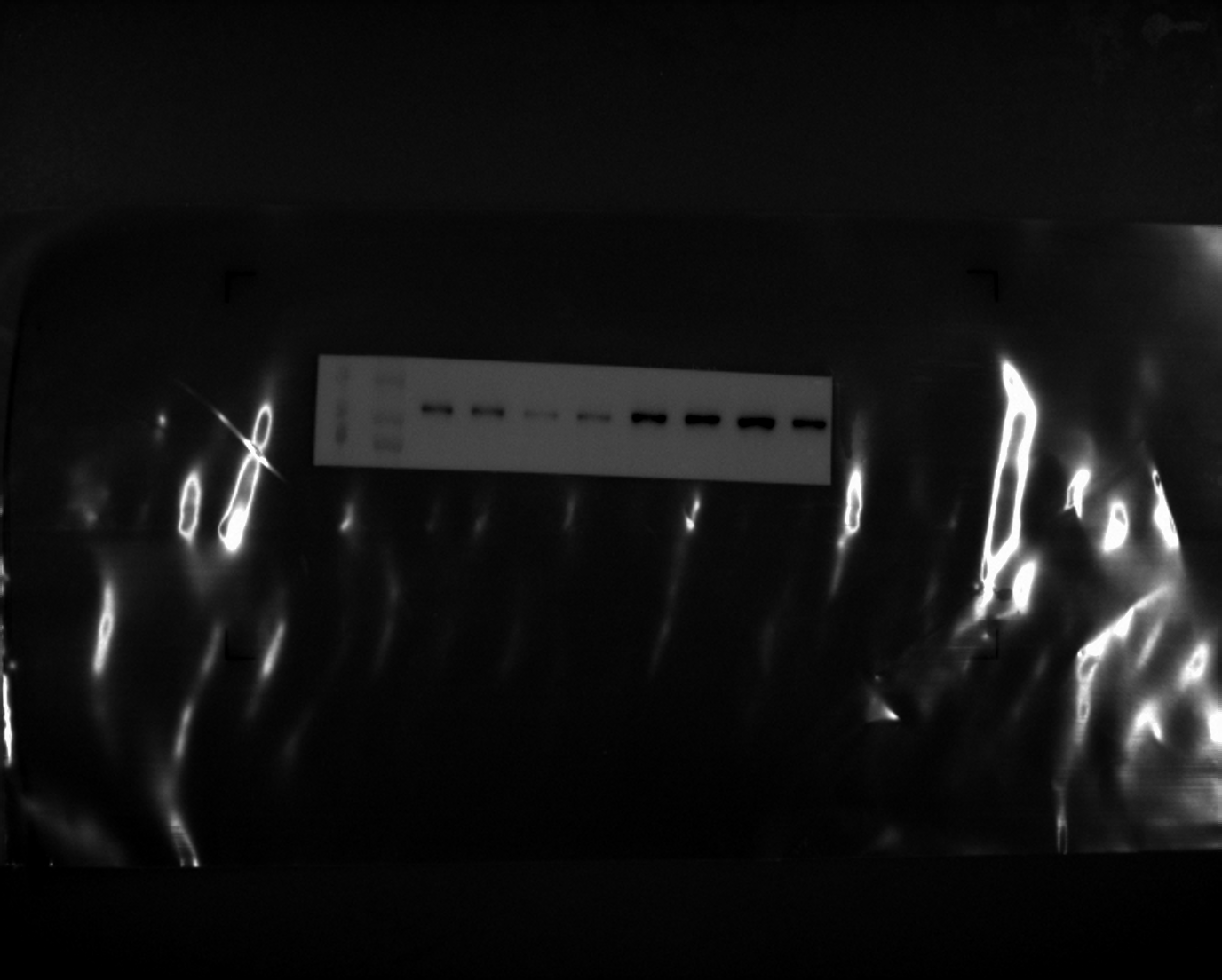

Supplement: Supplementary file 5 — Supplementary Material 5 [file 12885_2023_11088_MOESM5_ESM.tif]

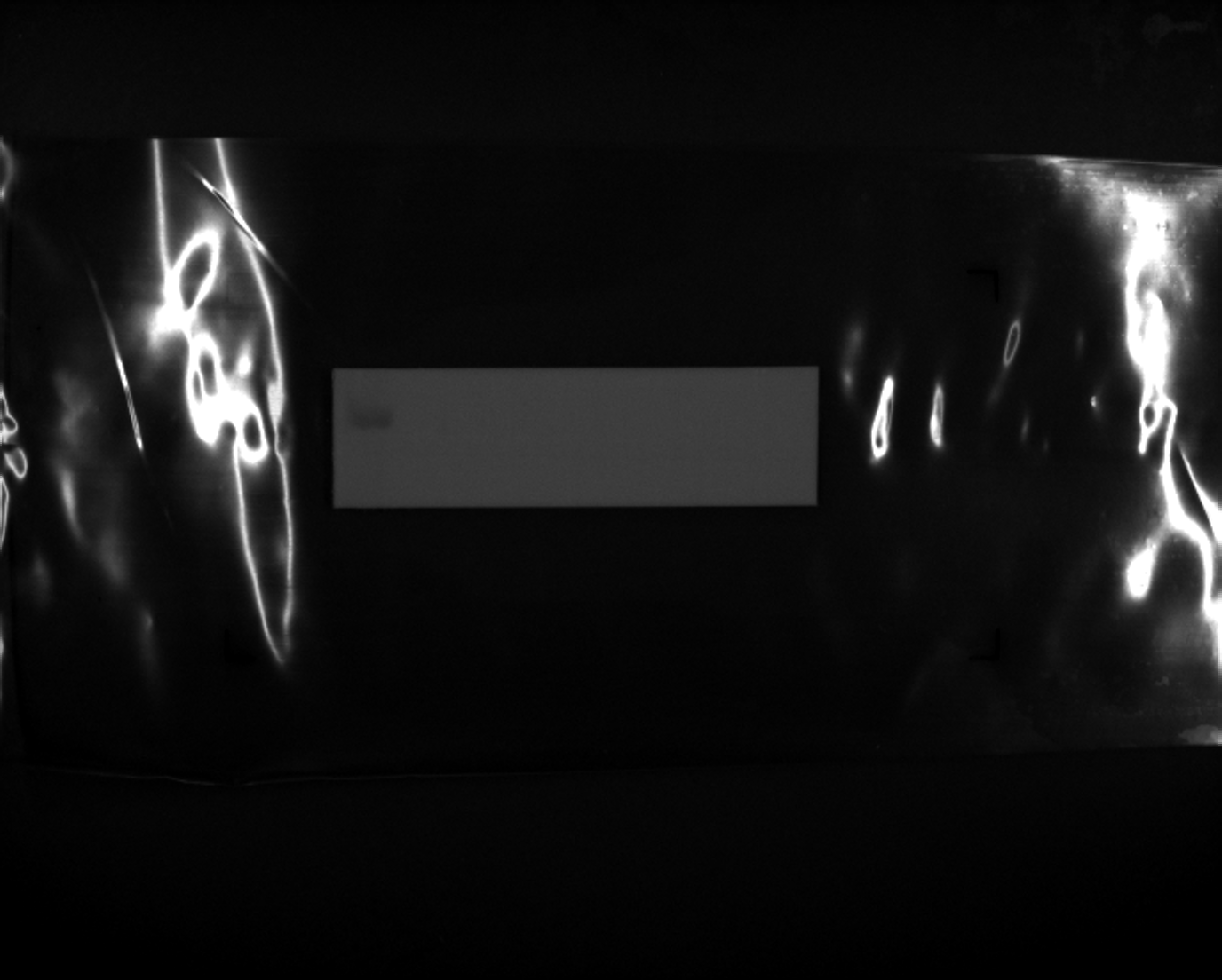

Supplement: Supplementary file 6 — Supplementary Material 6 [file 12885_2023_11088_MOESM6_ESM.tif]

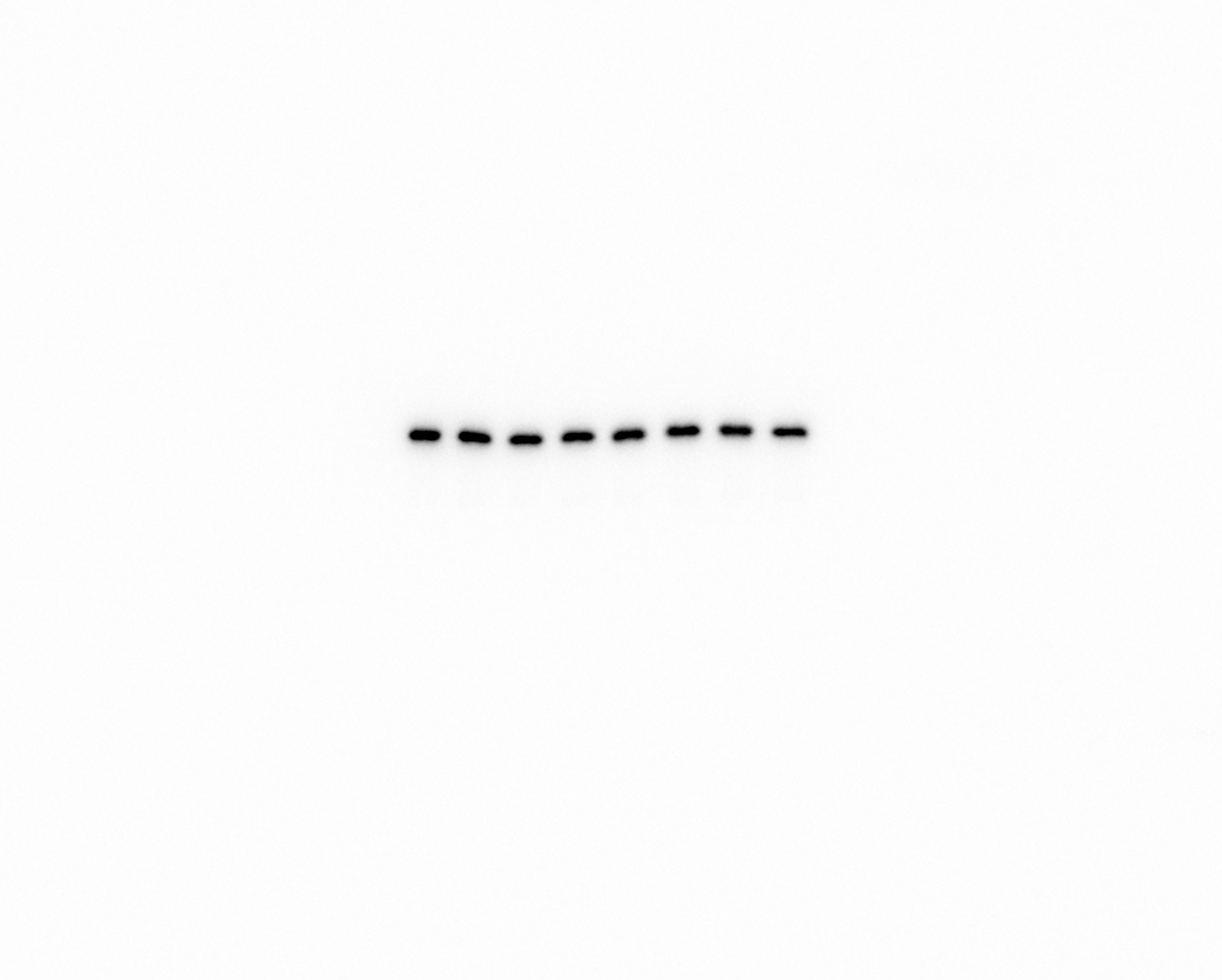

Supplement: Supplementary file 7 — Supplementary Material 7 [file 12885_2023_11088_MOESM7_ESM.tif]

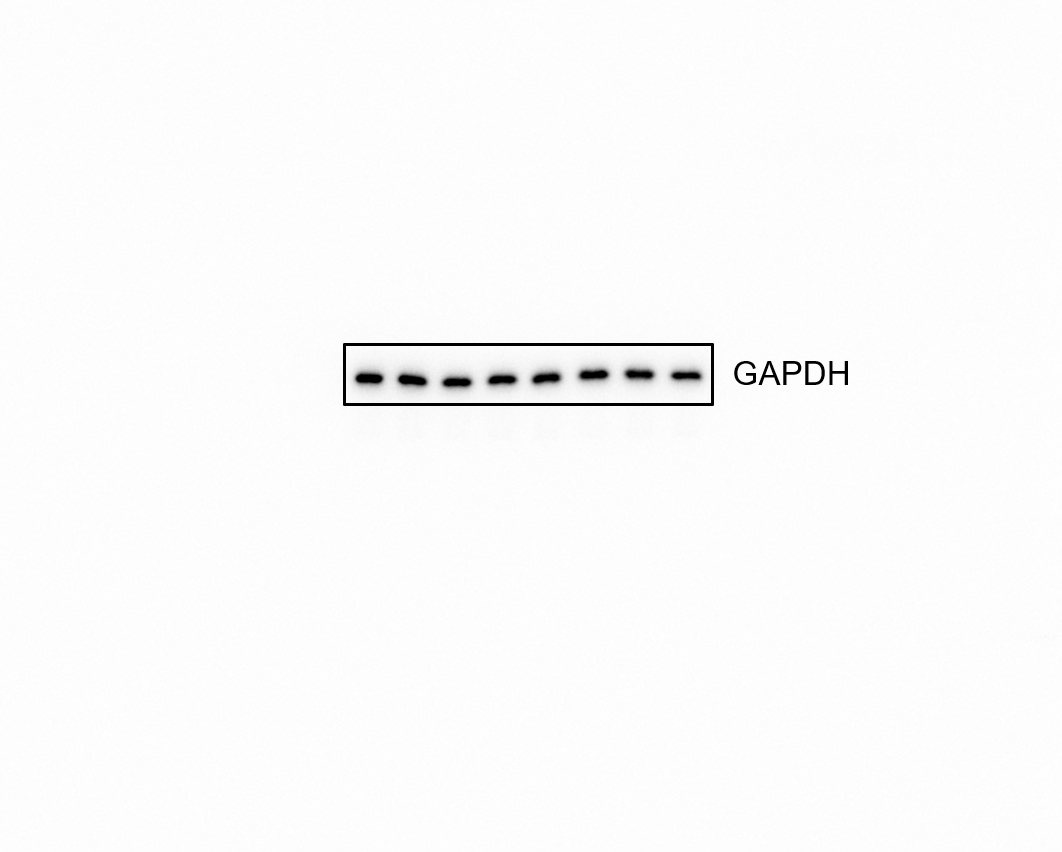

Supplement: Supplementary file 8 — Supplementary Material 8 [file 12885_2023_11088_MOESM8_ESM.tif]

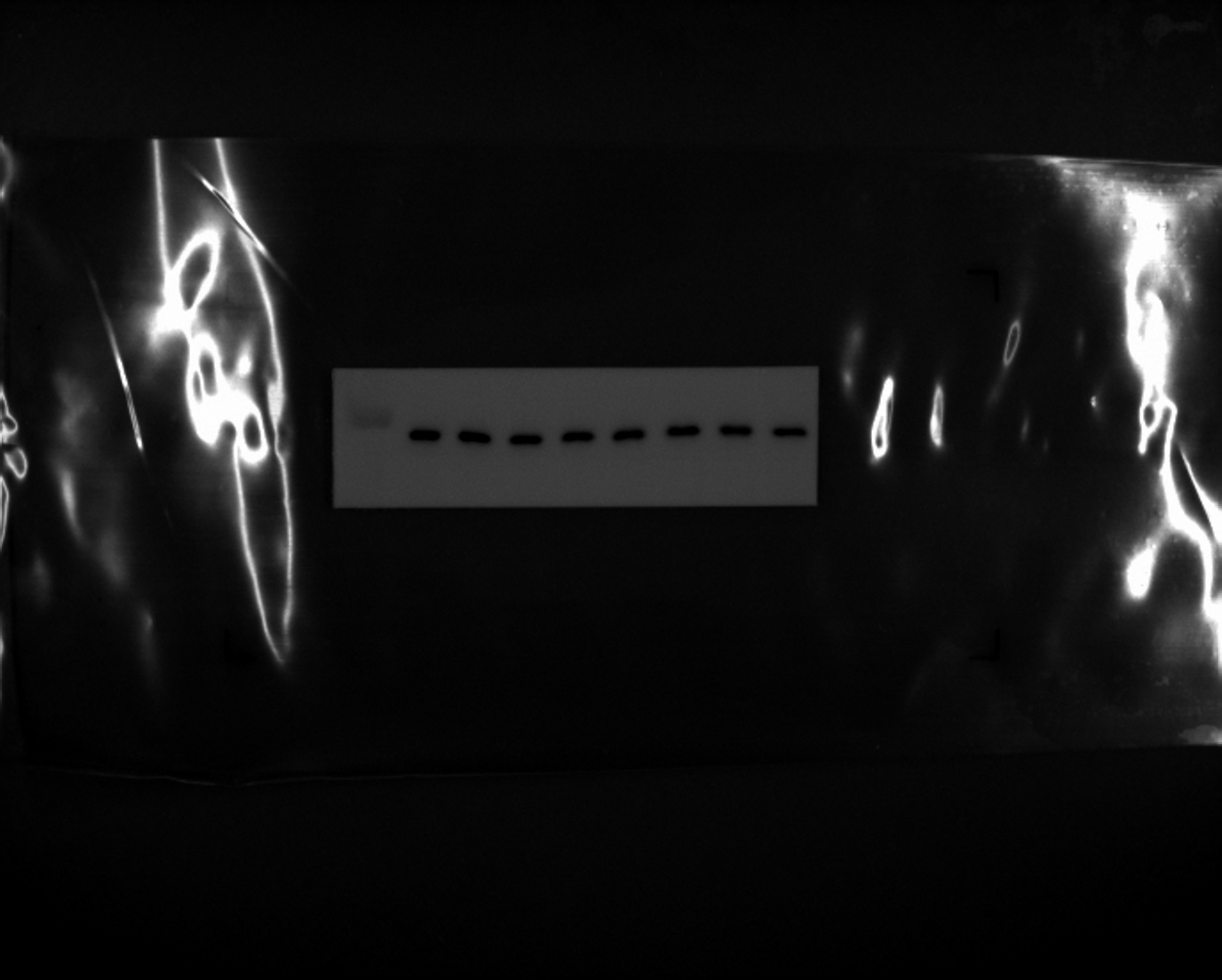

Supplement: Supplementary file 9 — Supplementary Material 9 [file 12885_2023_11088_MOESM9_ESM.tif]

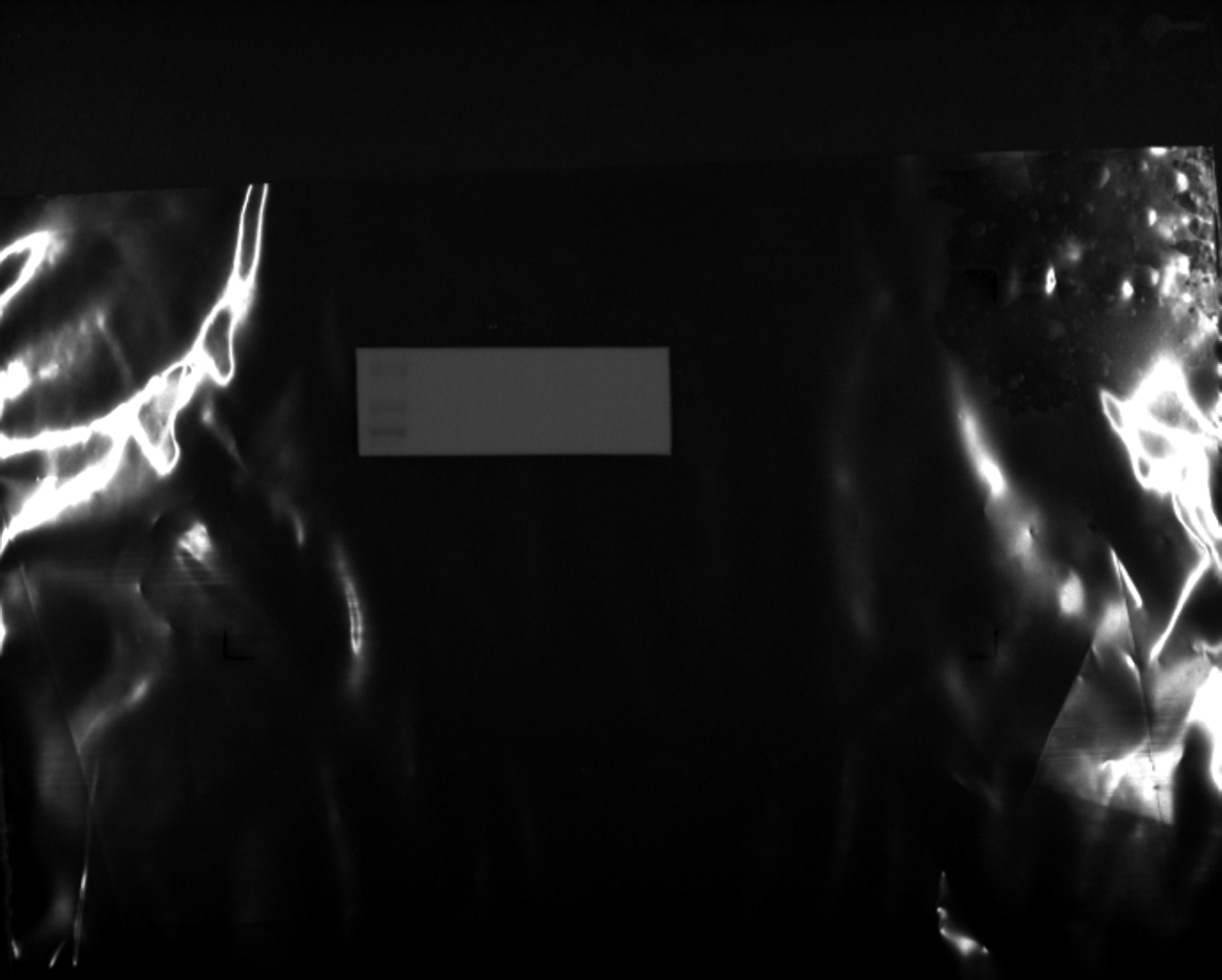

Supplement: Supplementary file 10 — Supplementary Material 10 [file 12885_2023_11088_MOESM10_ESM.tif]

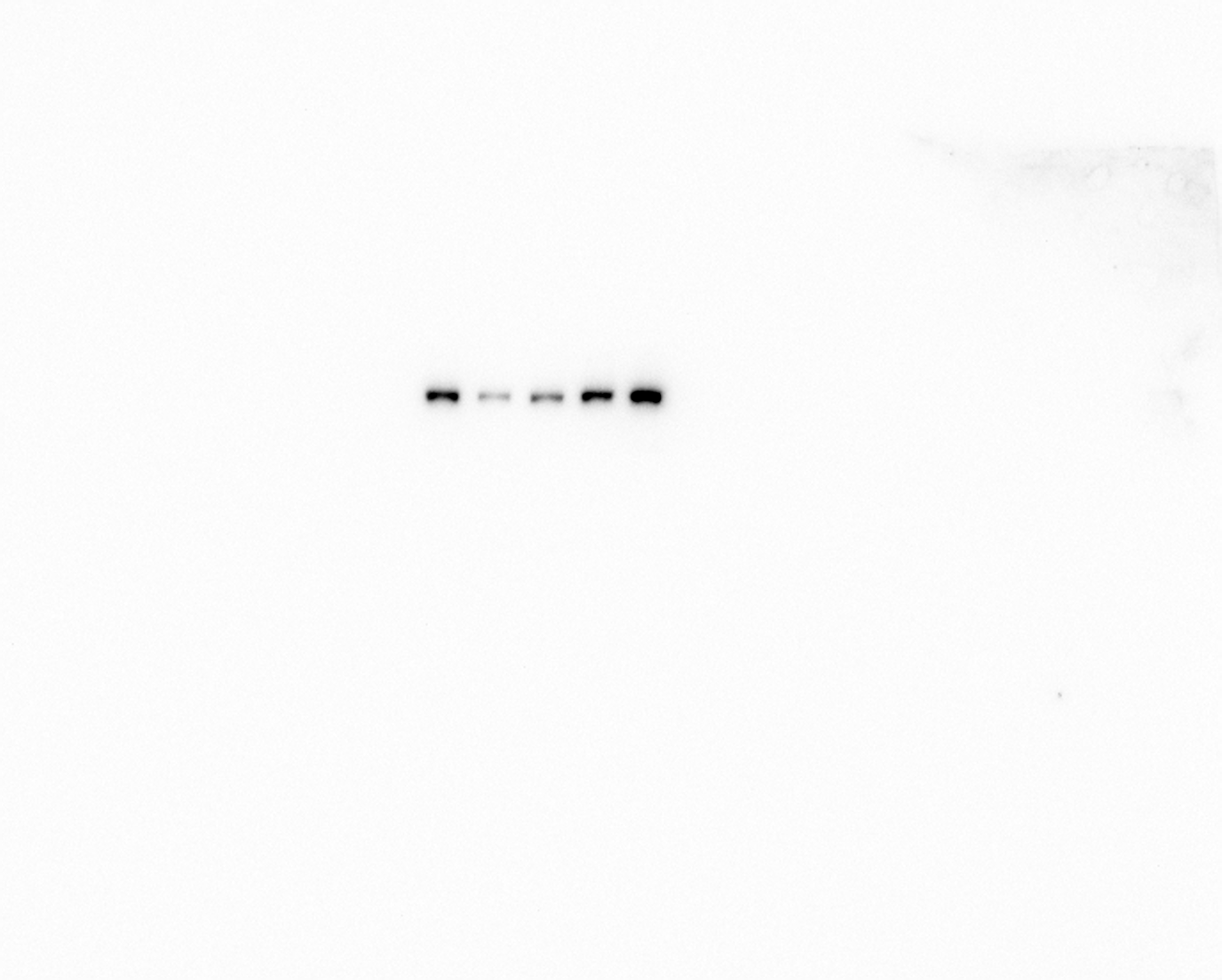

Supplement: Supplementary file 11 — Supplementary Material 11 [file 12885_2023_11088_MOESM11_ESM.tif]

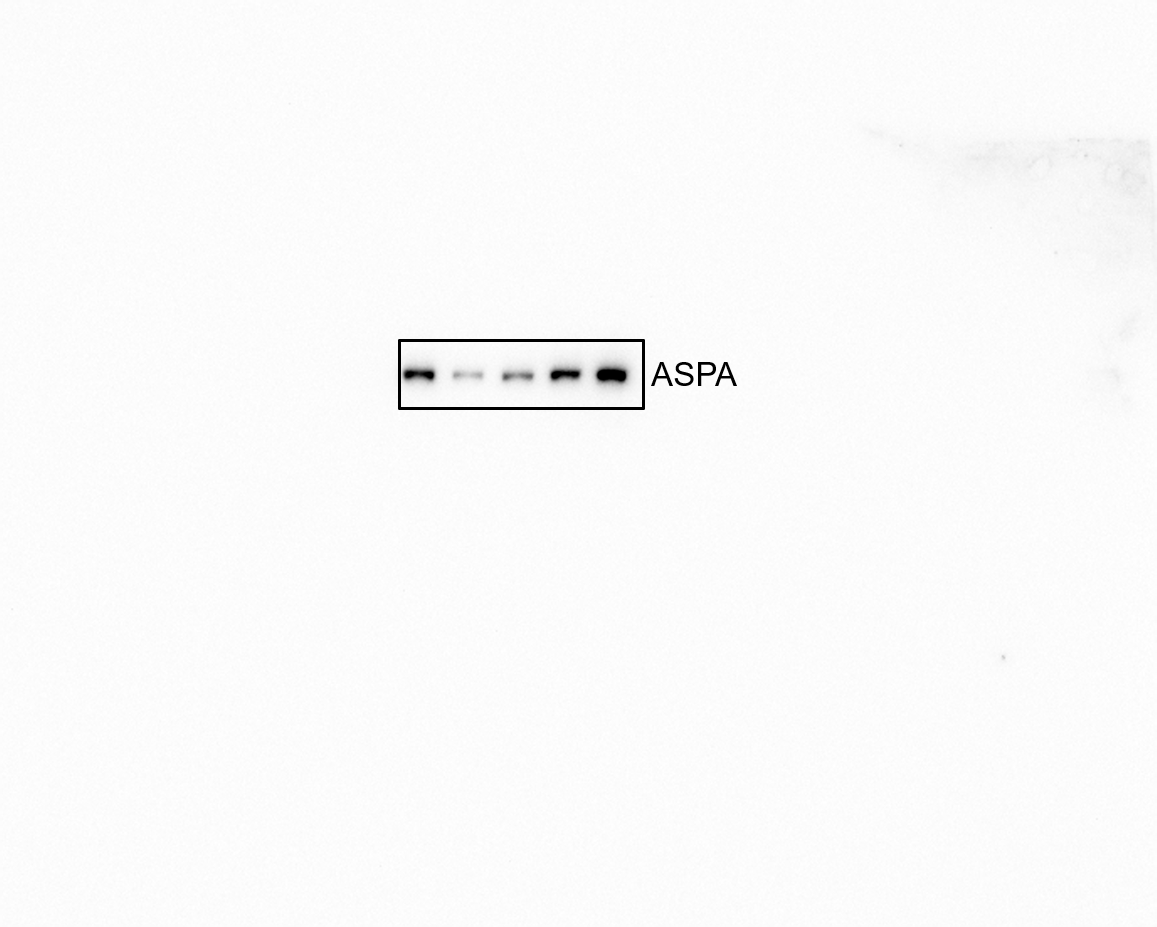

Supplement: Supplementary file 12 — Supplementary Material 12 [file 12885_2023_11088_MOESM12_ESM.tif]

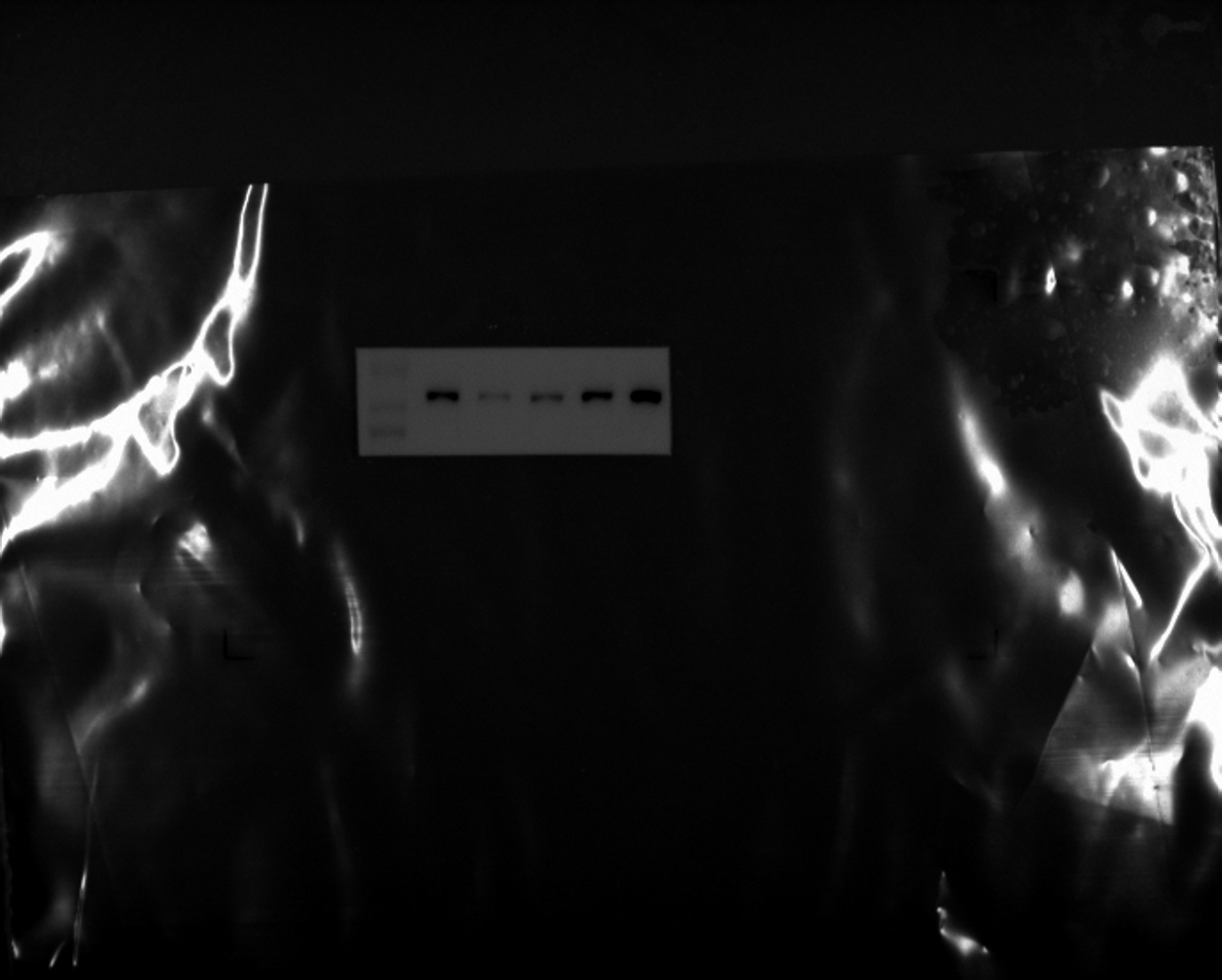

Supplement: Supplementary file 13 — Supplementary Material 13 [file 12885_2023_11088_MOESM13_ESM.tif]

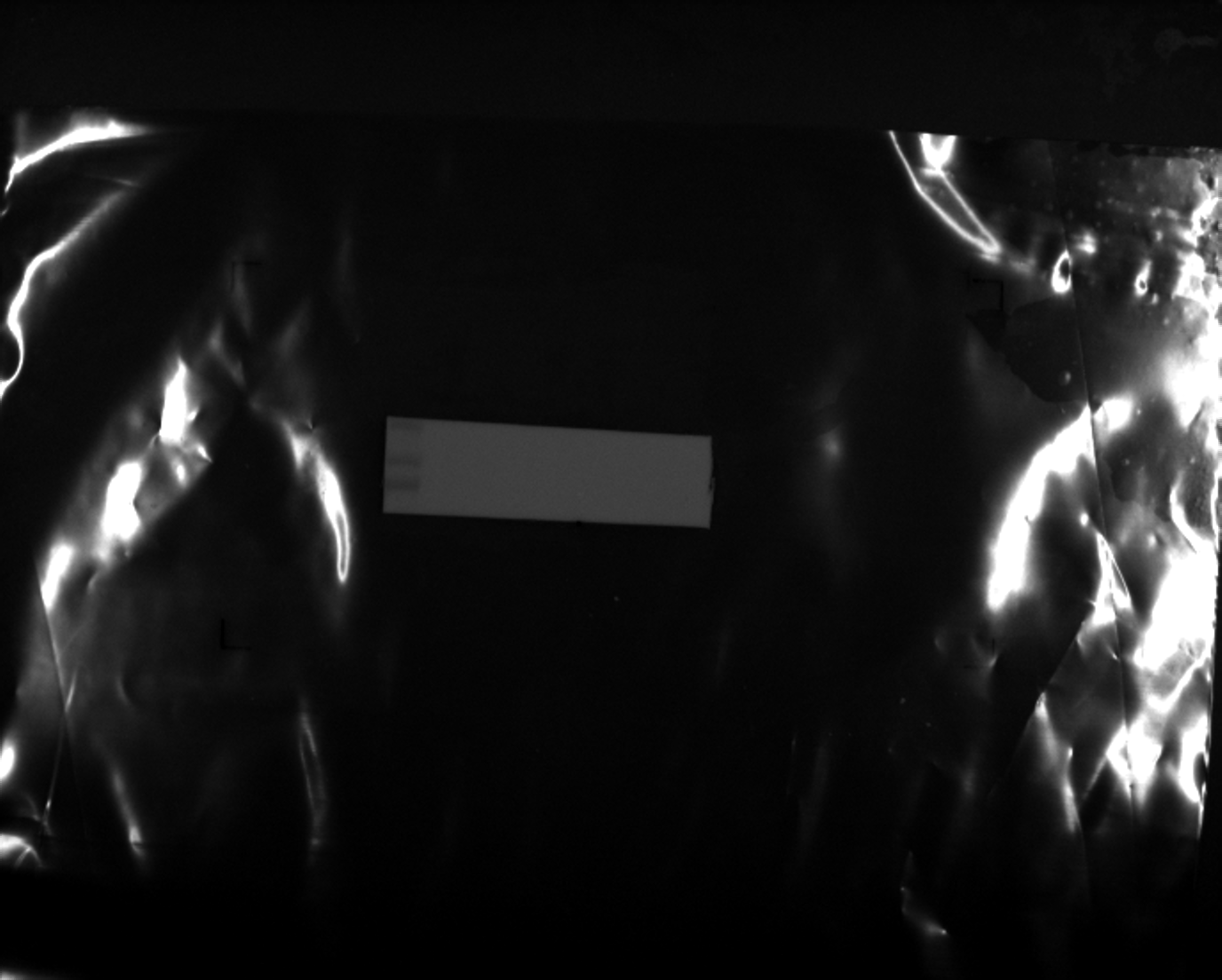

Supplement: Supplementary file 14 — Supplementary Material 14 [file 12885_2023_11088_MOESM14_ESM.tif]

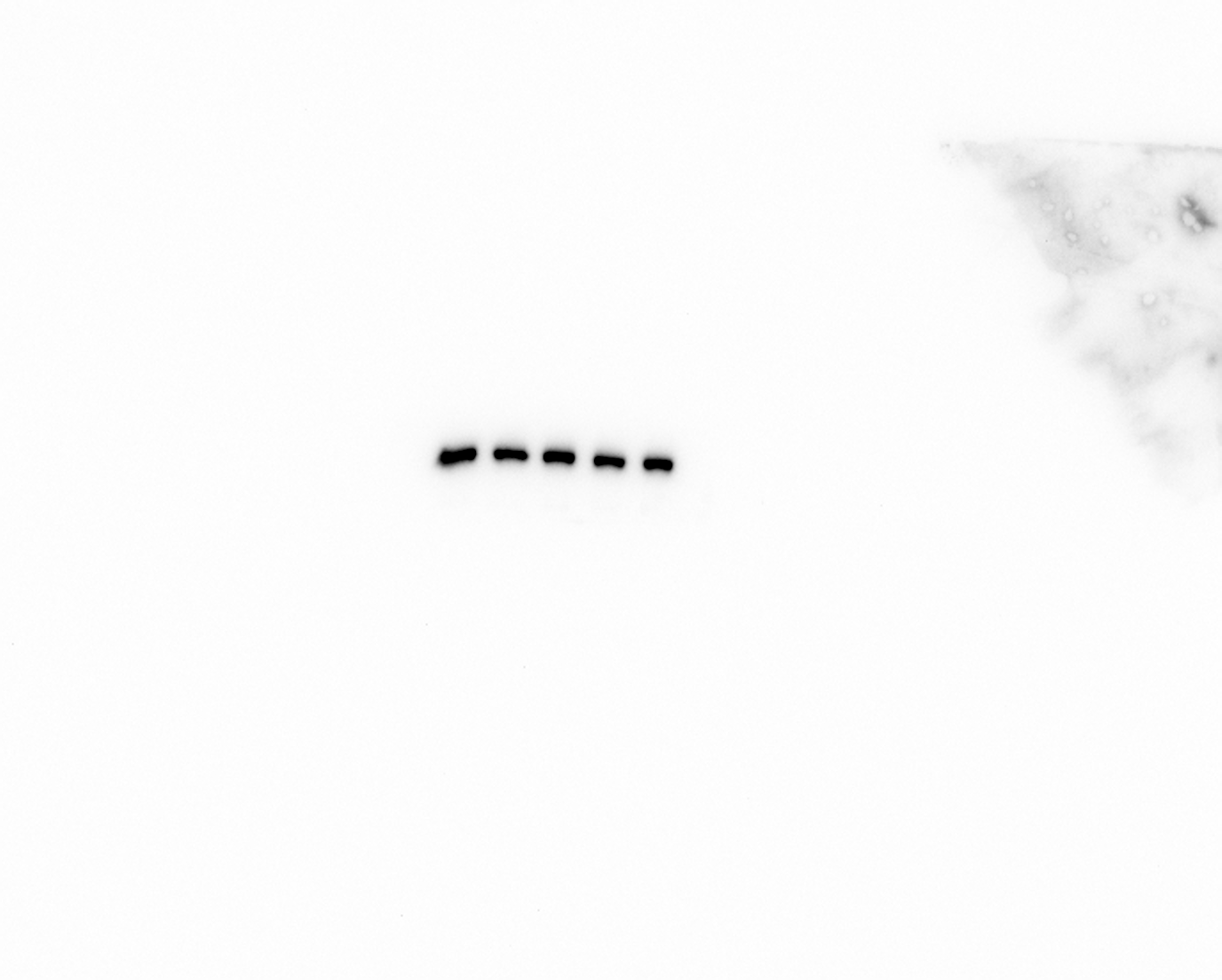

Supplement: Supplementary file 15 — Supplementary Material 15 [file 12885_2023_11088_MOESM15_ESM.tif]

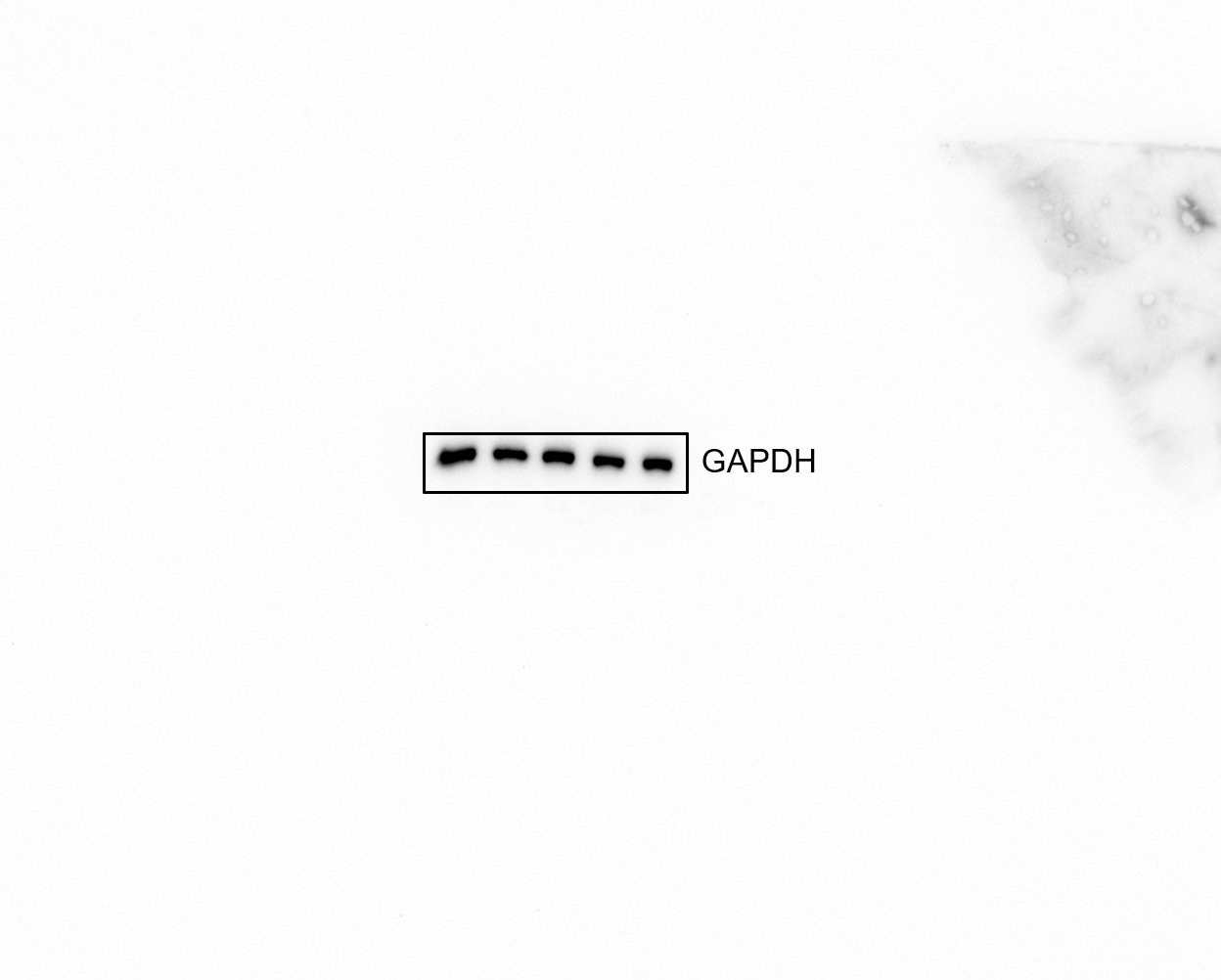

Supplement: Supplementary file 16 — Supplementary Material 16 [file 12885_2023_11088_MOESM16_ESM.tif]

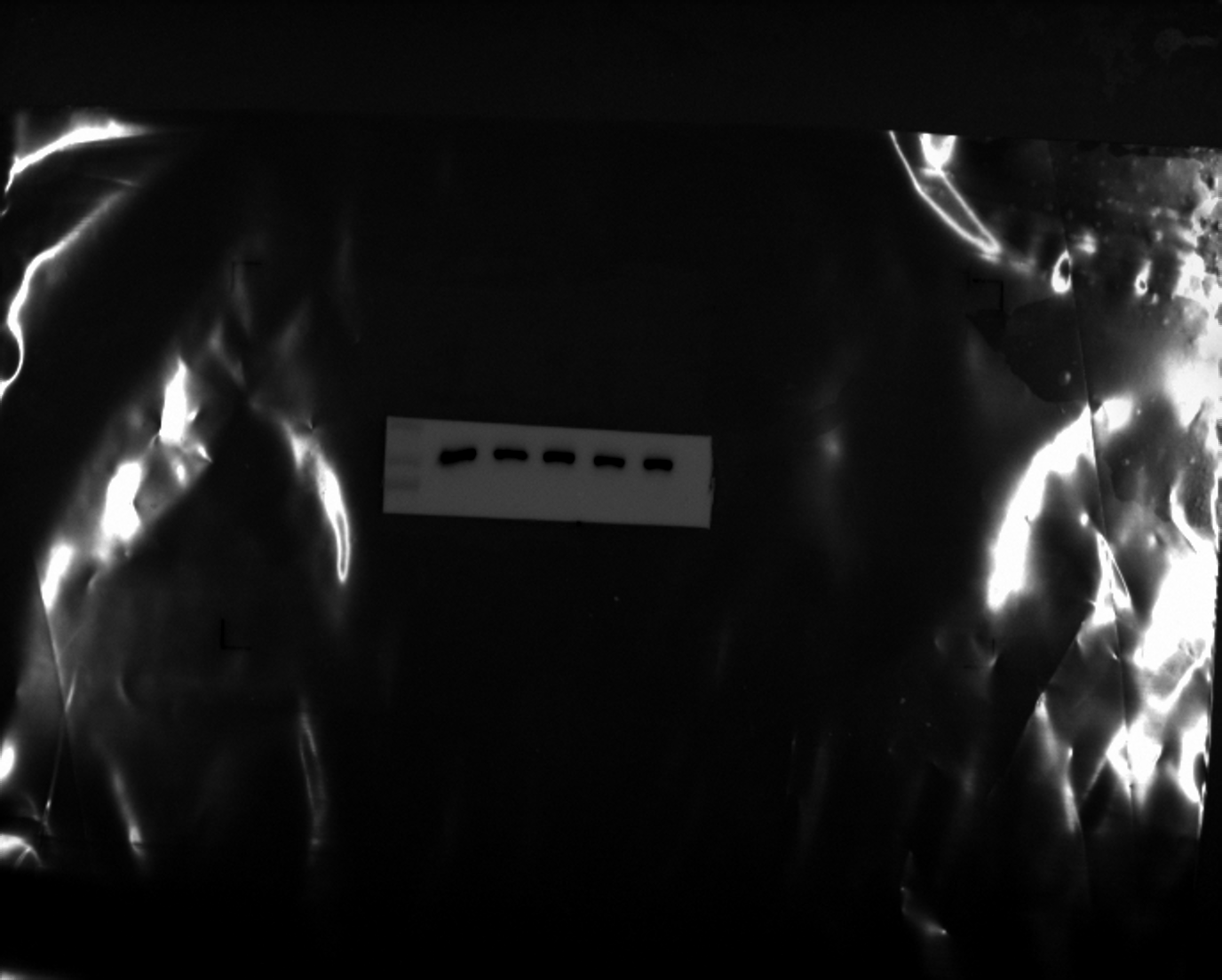

Supplement: Supplementary file 17 — Supplementary Material 17 [file 12885_2023_11088_MOESM17_ESM.tif]

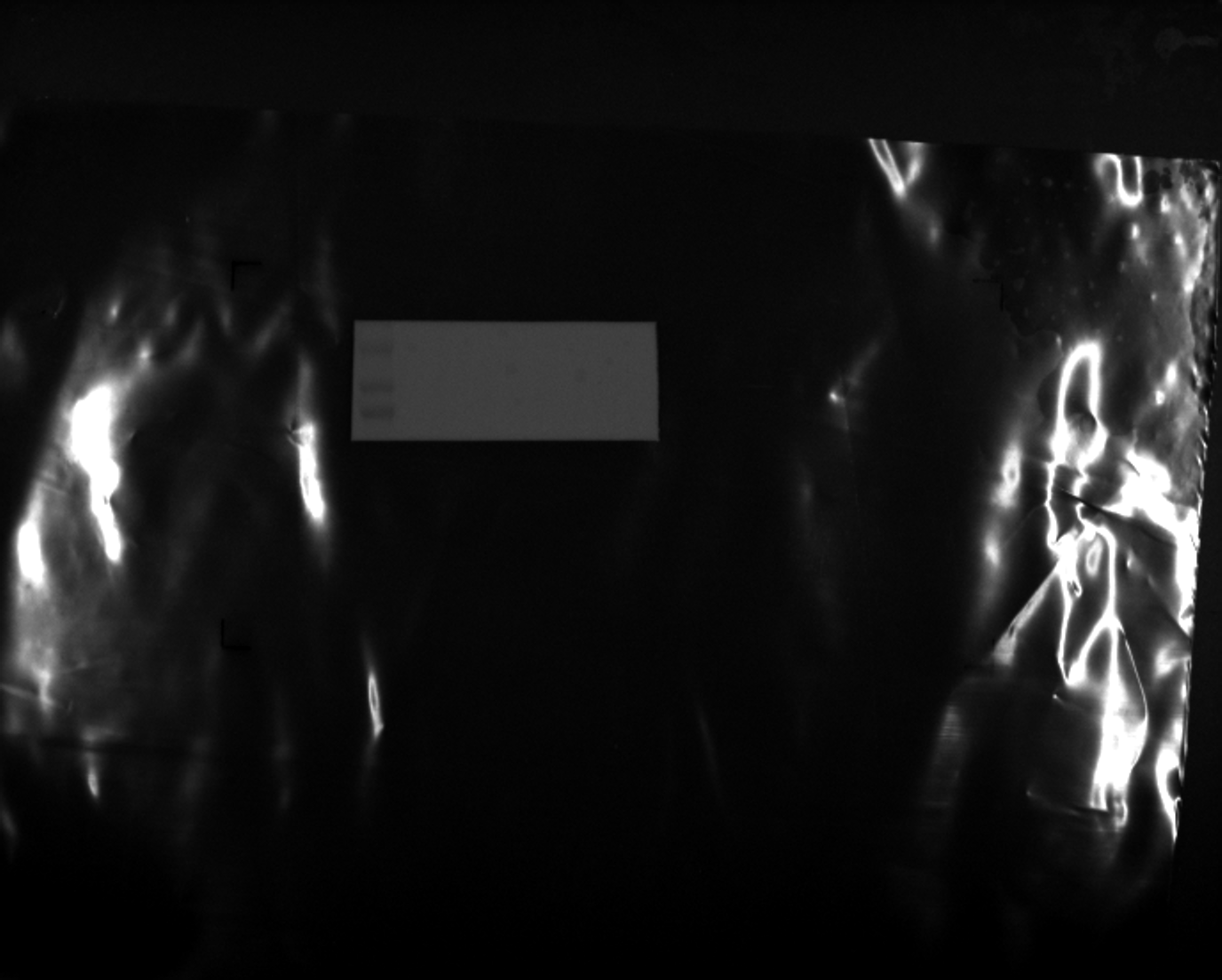

Supplement: Supplementary file 18 — Supplementary Material 18 [file 12885_2023_11088_MOESM18_ESM.tif]

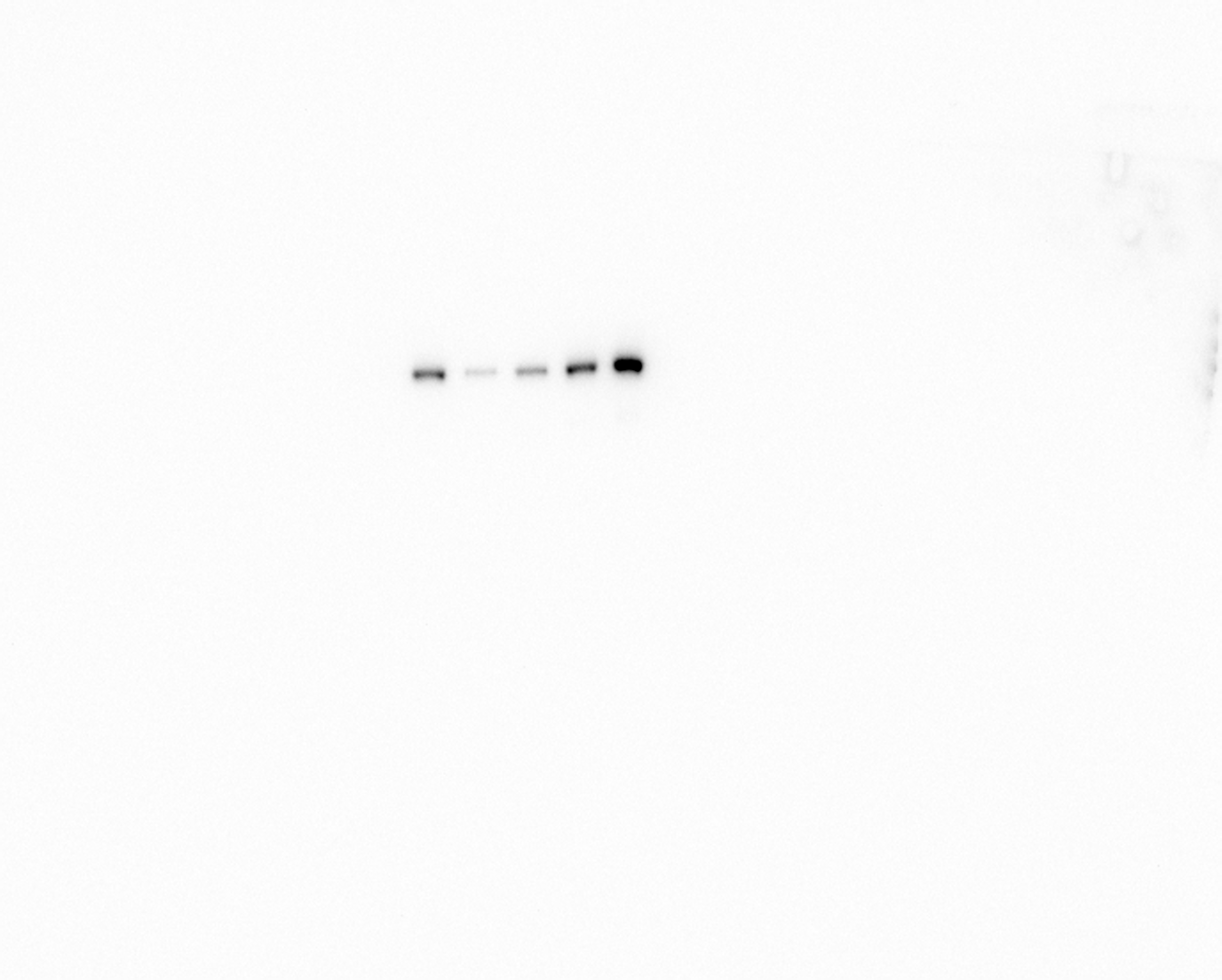

Supplement: Supplementary file 19 — Supplementary Material 19 [file 12885_2023_11088_MOESM19_ESM.tif]

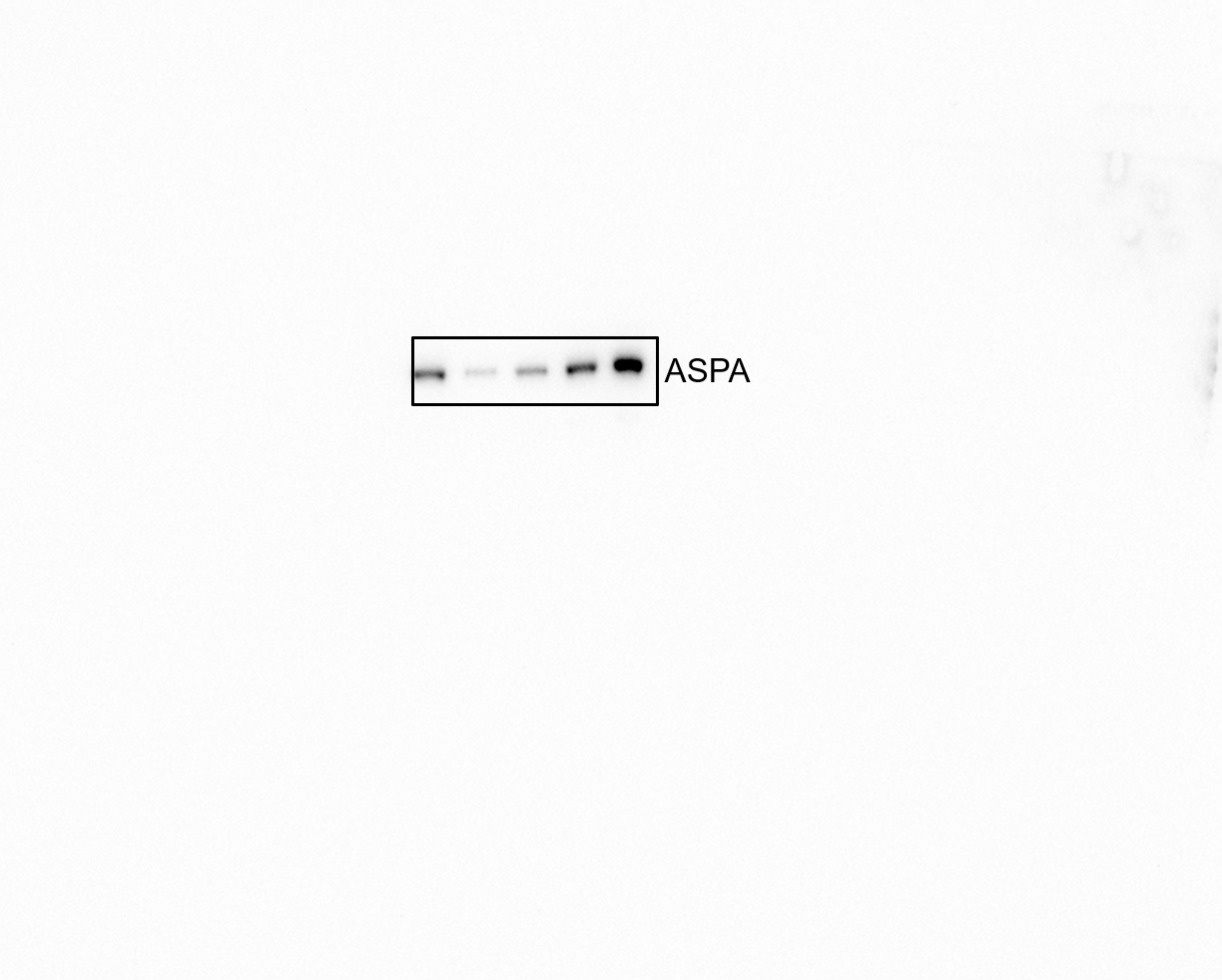

Supplement: Supplementary file 20 — Supplementary Material 20 [file 12885_2023_11088_MOESM20_ESM.tif]

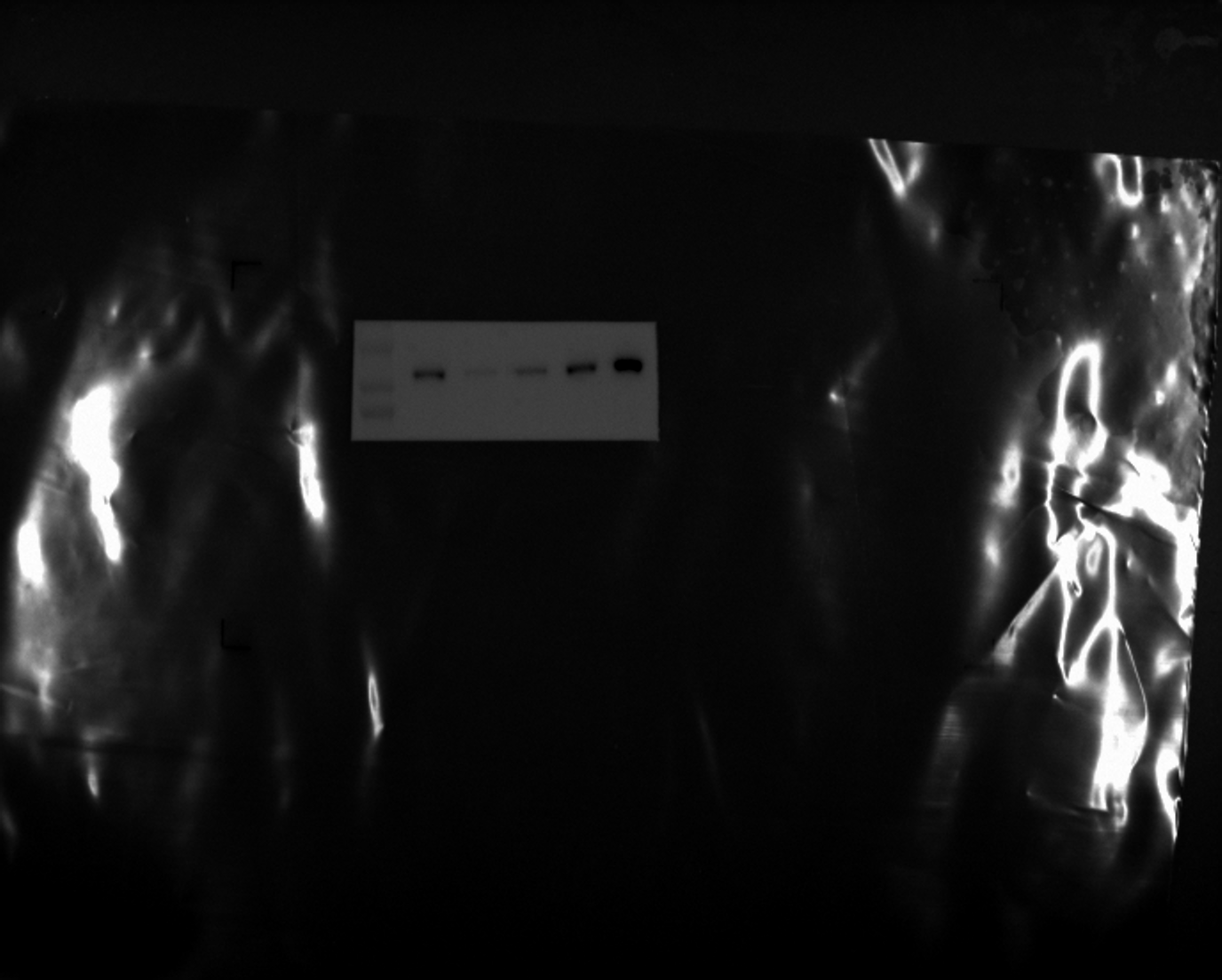

Supplement: Supplementary file 21 — Supplementary Material 21 [file 12885_2023_11088_MOESM21_ESM.tif]

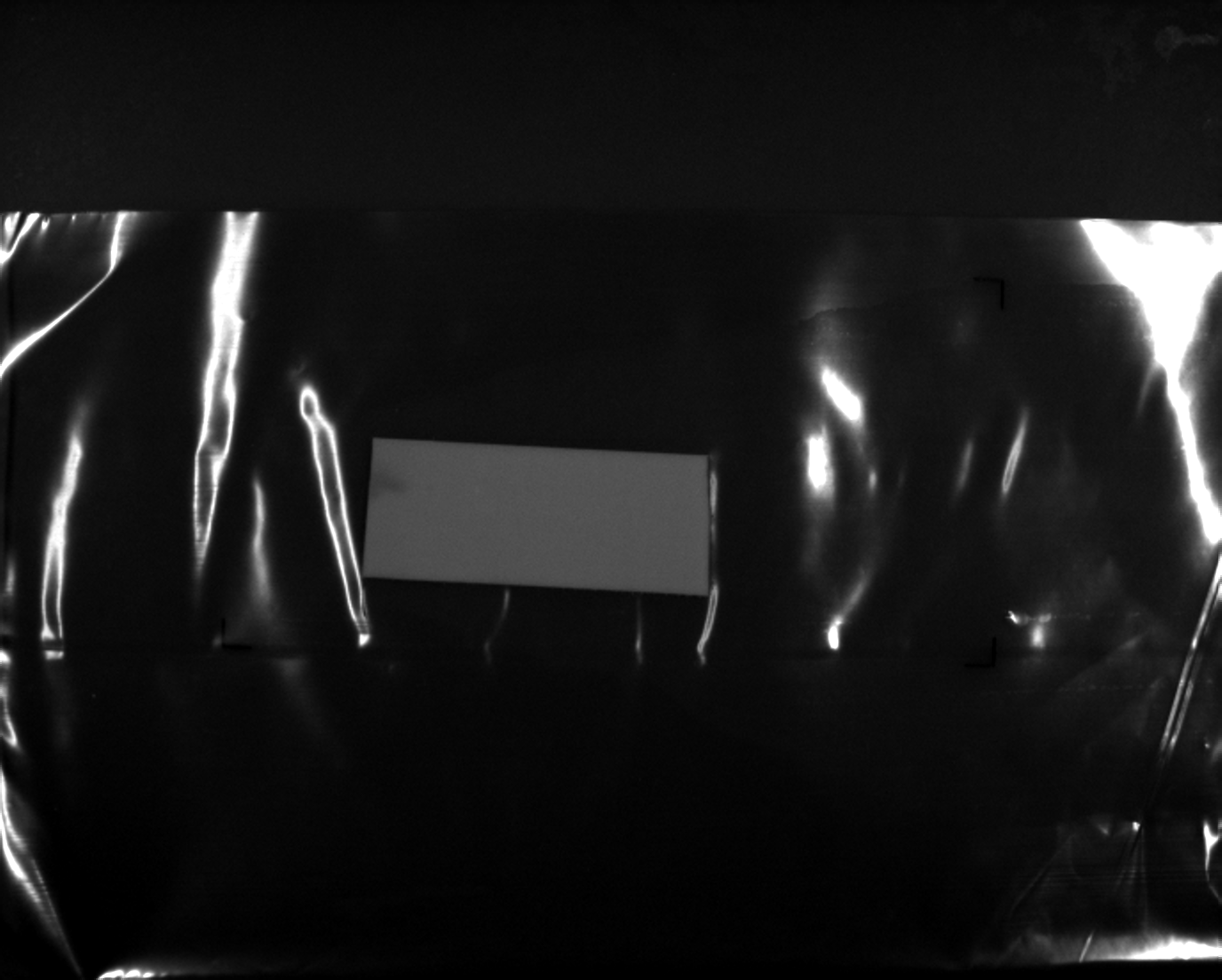

Supplement: Supplementary file 22 — Supplementary Material 22 [file 12885_2023_11088_MOESM22_ESM.tif]

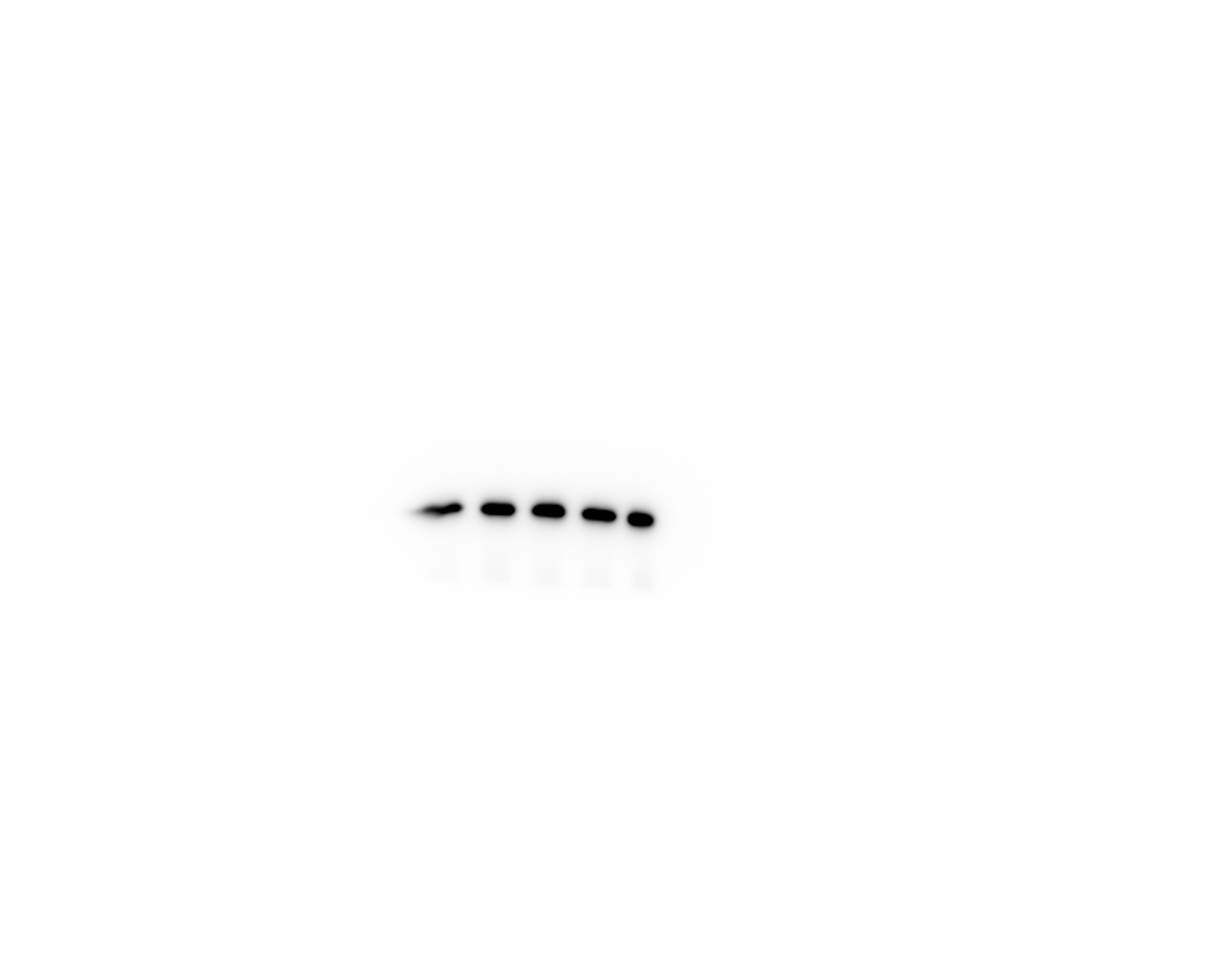

Supplement: Supplementary file 23 — Supplementary Material 23 [file 12885_2023_11088_MOESM23_ESM.tif]

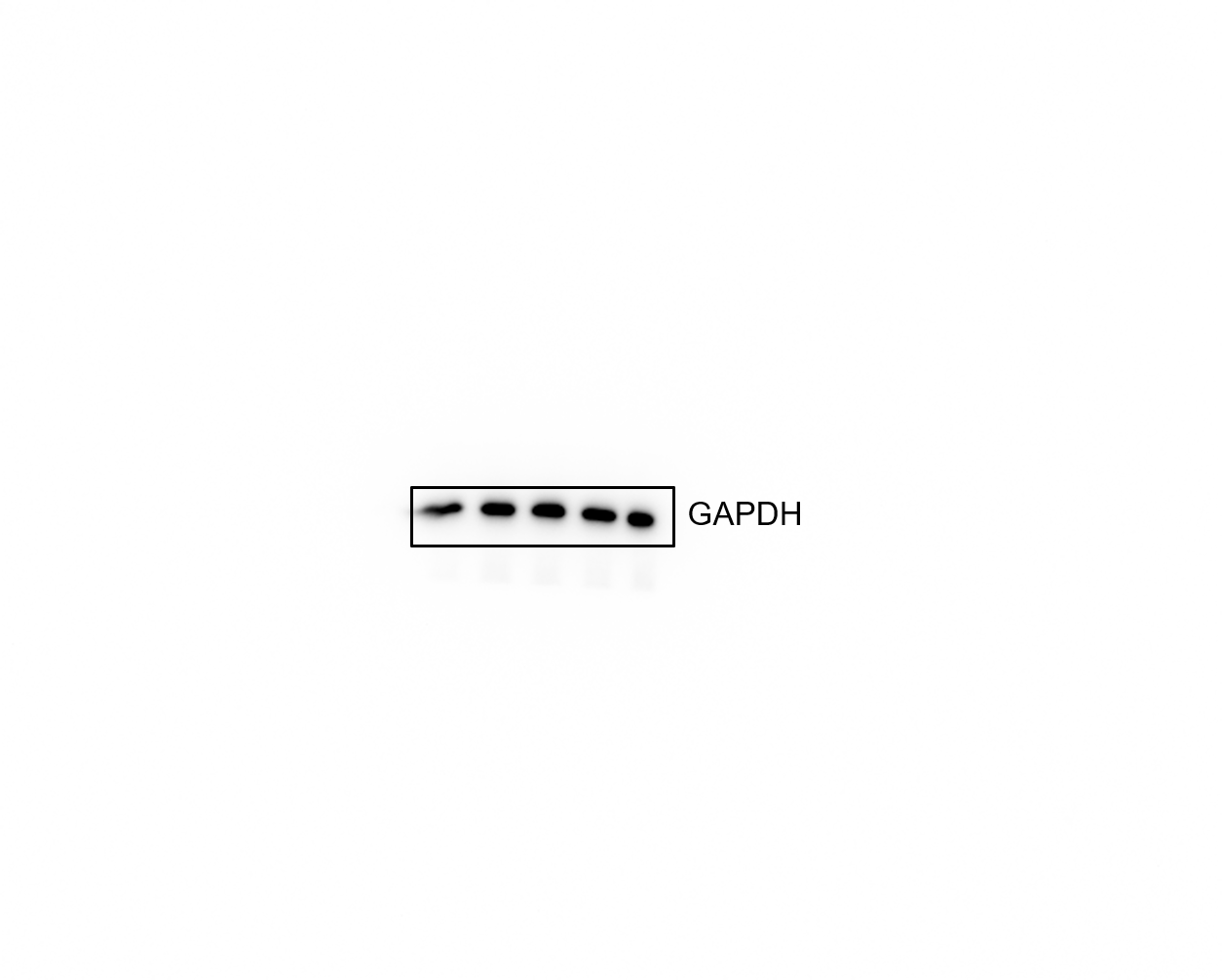

Supplement: Supplementary file 24 — Supplementary Material 24 [file 12885_2023_11088_MOESM24_ESM.tif]

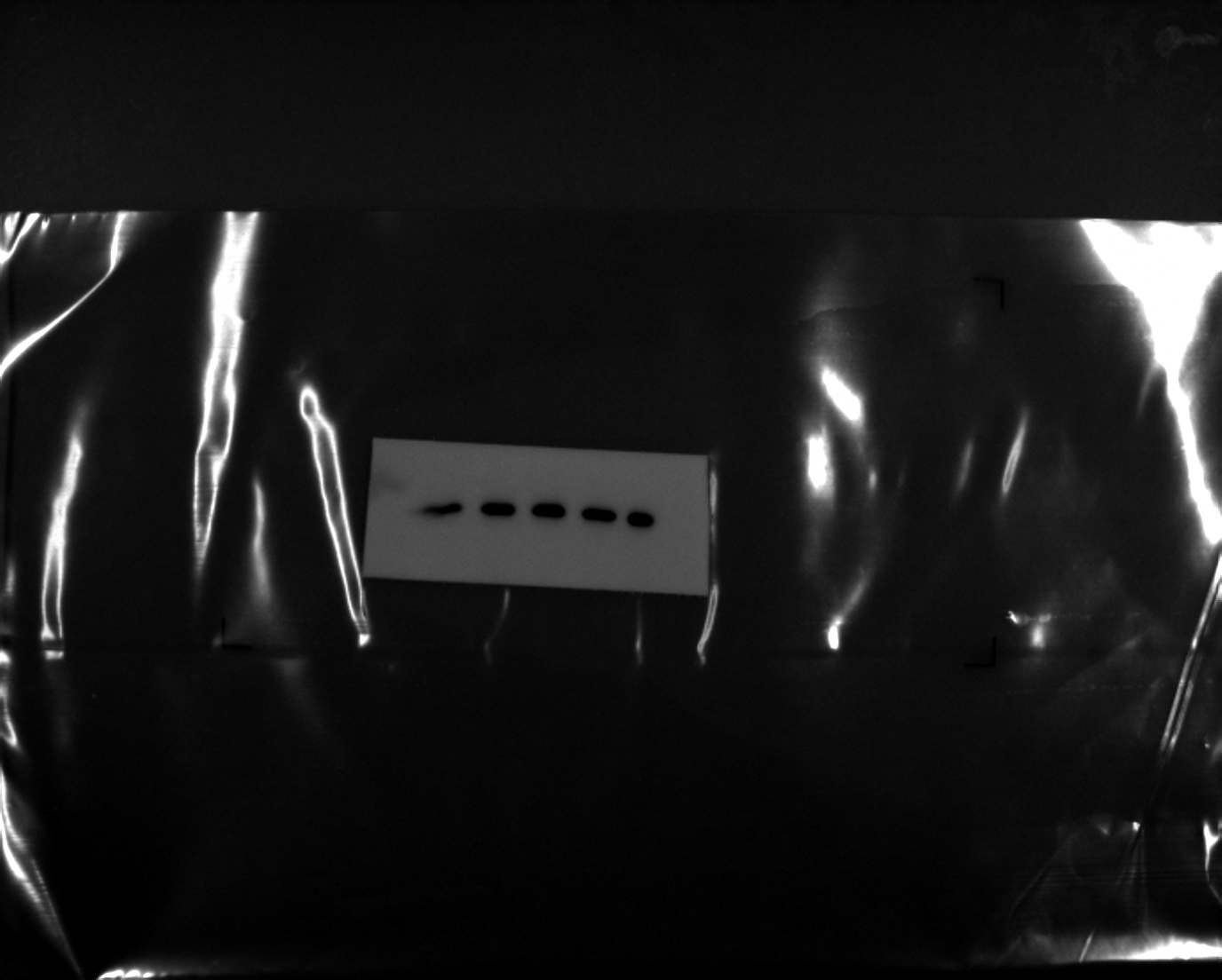

Supplement: Supplementary file 25 — Supplementary Material 25 [file 12885_2023_11088_MOESM25_ESM.tif]
